# Combined versus independent effects of exercise training and intermittent fasting on body composition and cardiometabolic health in adults: a systematic review and meta-analysis

**DOI:** 10.1186/s12937-023-00909-x

**Published:** 2024-01-06

**Authors:** Mousa Khalafi, Michael E. Symonds, Aref Habibi Maleki, Mohammad Hossein Sakhaei, Mahsa Ehsanifar, Sara K. Rosenkranz

**Affiliations:** 1 Department of Physical Education and Sport Sciences, Faculty of Humanities, University of Kashan, Kashan, Iran; 2 Centre for Perinatal Research, Academic Unit of Population and Lifespan Sciences, School of Medicine, University of Nottingham, Nottingham, NG7 2UH UK; 3 Department of Exercise Physiology and Corrective Exercises, Faculty of Sport Sciences, Urmia University, Urmia, Iran; 4 Department of Exercise Physiology, Faculty of Sport Sciences, University of Guilan, Guilan, Iran; 5 Department of Kinesiology and Nutrition Sciences, University of Nevada Las Vegas, Las Vegas, NV USA

## Abstract

**Introduction and aim:**

Exercise training (Ex) and intermittent fasting (IF) are effective for improving body composition and cardiometabolic health overweight and obese adults, but whether combining Ex and IF induces additive or synergistic effects is less well established. We therefore, performed a systematic review and meta-analysis to compare the combined versus independent effects of Ex and IF on body composition and cardiometabolic health in adults.

**Method:**

An electronic search was conducted in three main online databases including PubMed, Web of Science, and Scopus, from inception to March 9, 2023 for studies involving Ex plus IF trials versus standalone Ex and/or IF interventions in adults. Interventions had a duration of ≥ 2 weeks. Standardized (SMD) or weighted mean differences (WMD) and 95% confidence intervals were calculated in order to compare effects on body weight, body mass index (BMI), body fat lean body mass (LBM), visceral fat, and waist circumference. For cardiometabolic health, outcomes included fasting glucose, insulin, total cholesterol (TC), low-density lipoprotein cholesterol (LDL), triglycerides (TG), high-density lipoprotein cholesterol (HDL), systolic (SBP) and diastolic (DBP) blood pressure, and VO_2_max/peak.

**Results:**

Ex plus IF decreased body weight [WMD: -3.03 kg (95% CI: -3.44 to -2.61), *p* = 0.001], BMI [WMD: -1.12 kg.m^2^ (95% CI: -1.28 to -0.95), *p* = 0.001], body fat [SMD: -0.72 (95% CI: -1.23 to -0.21), *p* = 0.005], visceral fat [SMD: -0.34 (95% CI: -0.63 to -0.05), *p* = 0.01], and waist circumference [WMD: -2.63 cm (95% CI: -4.16 to -1.11), *p* = 0.001] more than Ex alone. However, changes in body composition and cardiometabolic health markers were not significantly different for Ex plus IF when compared with IF alone, with the exception of VO_2_max/peak [SMD: 0.55 (95% CI: 0.14 to 0.97), *p* = 0.009].

**Conclusion:**

We demonstrate that a combination of Ex and IF produces superior changes in body composition, but not in markers of cardiometabolic health when compared with Ex or IF alone. Ex plus IF could therefore be effective for weight and fat loss but has no additive or synergistic effects for other cardiometabolic health markers.

**Supplementary Information:**

The online version contains supplementary material available at 10.1186/s12937-023-00909-x.

## Introduction

Overweight and obesity are primary risk factors for the development of non-communicable chronic diseases including cardiovascular and metabolic diseases such as type 2 diabetes mellitus [1–3]. Although the etiology of obesity is complex, an imbalance between caloric intake and expenditure is a primary cause of obesity and subsequent co-morbid chronic diseases [4]. Obesity is associated with numerous complications including insulin resistance, low-grade inflammation, hypertension, dyslipidemia, and endothelial dysfunction, which all contribute to the development of cardiometabolic disease [3, 5–10]. With the worldwide epidemic of obesity [11], both pharmacological and non-pharmacological interventions are widely used in preventing and managing obesity related disease.

Non-pharmacological interventions including exercise training (Ex) and dietary modifications are initial treatment strategies for obesity and the prevention of co-morbid conditions [12–15]. Ex is an effective intervention and is associated with substantial cardiometabolic health benefits such as improved insulin resistance, lipid profiles, and blood pressure [16, 17]. Regardless of exercise type, various meta-analyses have suggested that Ex improves cardiometabolic health in overweight and obese adults with co-morbid conditions, independent of sex and age. Dietary interventions, primarily caloric restriction, are also effective [18–21], but have the potential to negatively affect muscle mass. In recent years, intermittent fasting (IF) has become an alternative and popular dietary intervention, including different eating patterns such as alternate-day fasting (ADF), 5 plus 2 diets, and time-restricted eating (TRE). Regardless of type, IF is effective in reduce body weight and fat mass, and is also associated with improvements in cardiometabolic health. In this regard, several meta-analyses have confirmed that IF is effective for improving lipid profiles, glycemic markers, and blood pressure [22–25].

Despite the beneficial effects of Ex and IF dietary interventions on weight loss and obesity management, the combination of exercise and dietary interventions appears to elicit larger effects as compared to exercise or dietary interventions alone [15, 26–30].. Several meta-analyses have suggested that combined Ex and dietary interventions may be more effective than standalone exercise or dietary interventions for improving body composition, inflammation, glycemic markers, and lipid profiles, [15, 26–30]. However, to date, no comprehensive meta-analysis has investigated the combined versus independent effects of Ex and IF interventions. Therefore, we completed a systematic review and meta-analysis to determine whether Ex combined with IF, compared with standalone Ex or IF interventions, has further beneficial effects on body composition and cardiometabolic health markers in adults.

## Method

The current systematic review and meta-analysis was conducted and written in accordance with the Preferred Reporting Items for Systematic Reviews and Meta-analyses (PRISMA) guidelines, and followed the additional guidance provided by the Cochrane Handbook of Systematic Reviews of Interventions. The systematic review and meta-analysis was pre-registered in the International prospective register of systematic reviews (PROSPERO; ID: CRD42023459841).

### Data sources and search strategy

A comprehensive electronic literature search was conducted in three main online databases including PubMed, Web of Science, and Scopus. The search was performed from inception to March 9, 2023 using the following key words: ("time-restricted feeding" OR "time restricted feeding" OR "time-restricted eating" OR "time restricted eating" OR "time-restricted diet" OR "time restricted diet" OR "time-restricted fasting" OR "time restricted fasting" OR "intermittent fasting" OR "intermittent energy restriction" OR "alternate fasting" OR "periodic fasting" OR "reduced meal frequency" OR "alternate-day fasting") and (exercise OR "exercise training" OR "physical activity" OR "aerobic training" OR "aerobic exercise" OR "resistance training" OR "resistance exercise" OR "combined training" OR "combined exercise" OR "concurrent training" OR "concurrent exercise" OR "interval training" OR "interval exercise"). Relevant key words were combined with the Boolean operators OR/AND. When available in databases, filters including human, English language, and journal were applied. In addition, manual searches of reference lists of all included studies and follow on searches in Google Scholar were performed to make sure that no eligible studies were missed. Complete search strategy details are summarized in Supplementary Table 1. The searches were conducted independently by two authors (M H S and A H M) and any disagreements were resolved by discussion with another author (M Kh).

### Study selection and inclusion and exclusion criteria

The following inclusion criteria were applied based on the Population, Intervention, Comparison, Outcomes, and Study Design (PICOS). For the population, studies of participants with overweight and obesity or hidden obesity and ages ≥ 18 years, and healthy individuals regardless of biological sex, were included. There were no limitations for participant health status, and therefore overweight and obese adults with and without co-morbid conditions were included. For intervention, studies that included combined Ex and IF trials with intervention durations ≥ 2 weeks were included. There were no limitations regarding mode, intensity, frequency, or time of exercise. For IF, interventions included ADF, TRE, and Ramadan diurnal intermittent fasting (RIF). For comparison, studies involving standalone Ex and/or IF interventions were included. For outcomes, studies were included when results were reported for body composition (body weight, BMI, bod fat including fat mass or body fat percentage, LBM, waist circumference and visceral fat); glycemic markers (fasting glucose, fasting insulin and insulin resistance); fasting lipid profiles (total cholesterol (TC), low-density lipoprotein cholesterol (LDL), triglycerides (TG), high-density lipoprotein cholesterol (HDL)); and blood pressure (systolic (SBP) and diastolic (DBP) and VO_2max/peck_. For study design, randomized trials comparing combined Ex IF versus either standalone Ex and/or IF interventions were included. Further inclusion criteria were: articles written in the English language, and peer-reviewed articles. Exclusion criteria were: non-original studies such as reviews and non-randomized trials, and studies including participants without overweight and obesity, and studies involving trained or athletic populations. Study selection was performed by two independent authors (M H S and A H M) and any disagreements were resolved by discussion with another author (M Kh). All retrieved studies were exported into EndNote (version 20.2.1) and duplicates were recorded were removed. The remaining studies were screened against the inclusion and exclusion criteria in two steps; 1) based on title and abstract, and 2) based on full-text.

### Data extraction and synthesis

For each eligible study, two authors independently extracted the following data, and any disagreements were resolved by discussion with another author (M Kh): (1) study characteristics including study design and sample size; (2) participant characteristics including age, biological sex, BMI, health status; (3) Ex characteristics including mode, intensity, frequency, and duration; (4) IF characteristics including type (ADF, TRE, or RIF) and duration; and (5) outcome variables including body weight, BMI, body fat (fat mass)or body fat percentage if fat mass was not available), visceral fat mass, lean body mass (LBM or fat-free mass (FFM) if LBM was not (available), waist circumference, fasting glucose, insulin and insulin resistance, fasting TC, LDL, TG, HDL, SBP and DBP, and VO_2_max/peak. In addition, other relevant data required for calculating effect sizes, including means and standard deviations (SDs) or mean changes and their SDs, and sample sizes for each outcome were extracted. When required, these data were extracted from figures using Getdata Graph Digitizer software. In addition, when required, relevant data were calculated from standard errors, medians, and interquartile ranges [31–33]. For lipid profiles, data were expressed in milligrams per deciliter (mg/dL), and when required mmol/L values were converted to mg/dL with the conversion factor 1 mg/dL = 0.0259 mmol/L (for TC, LDL, and HDL) and 1 mg/dL = 0.0113 mmol/L (for TG) [34]. For studies where data were not available or were not able to be extracted from figures, the corresponding authors were contacted [35], but no response was received. Meta-analyses were performed when there were three or more intervention studies for each variable.

### Quality assessment

Quality assessments were conducted for all included studies according to the Physiotherapy Evidence Database (PEDro) Scale, a valid measure of the methodologic quality of clinical trials [36]. The items on this assessment include: (1) eligibility criteria specified, (2) random allocation of participants, (3) allocation concealment, (4) groups similar at baseline, (5) blinding of participants, (6) blinding of intervention groups, (7) blinding of assessors, (8) outcome measures assessed in more than 85% of participants, (9) intervention to treat analysis, (10) reporting of between-group statistical comparisons and point measures, and (11) measures of variability reported for mean effect. Each item is scored as either present () or absent (x). Two authors (M H S and A H M) independently assessed the quality of each study, and any disagreements were resolved by discussion with another author (M Kh).

### Statistical analysis

Two separate analyses were conducted to calculate the effect sizes for determining the effects for the following on the main outcomes: (1) combined Ex and IF versus Ex only, and (2) combined Ex and IF versus IF only. The standardized mean differences (SMDs) or weighted mean differences (WMDs) and 95% confidence intervals were calculated using random effect models to determine measures of intervention effectiveness. Interpretation for SMDs was according to the Cochrane guidelines, with 0.20–0.49, 0.50–0.79, and ≥ 0.8 indicating small, medium, and large effect sizes, respectively. Heterogeneity was quantified using I^2^ and decomposed Q-statistics, where I^2^ was interpreted according to Cochrane guidelines, with 25%, 50%, and 75% indicating low, moderate, and high heterogeneity, respectively. Publication bias was assessed using visual interpretation of funnel plots with Egger’s tests as secondary determinants, where a p-value of < 0.10 indicated possible publication bias. In addition, the trim and fill method was used to correct the potential effects of publication bias when visual interpretation of funnel plots indicated publication bias. Sensitivity analyses were performed when 10 or more intervention arms were included in an analysis. All analyses and funnel and forest plots presented, were conducted using comprehensive meta-analysis (CMA) software version 3.

## Results

### Included studies

**Fig. 1:**
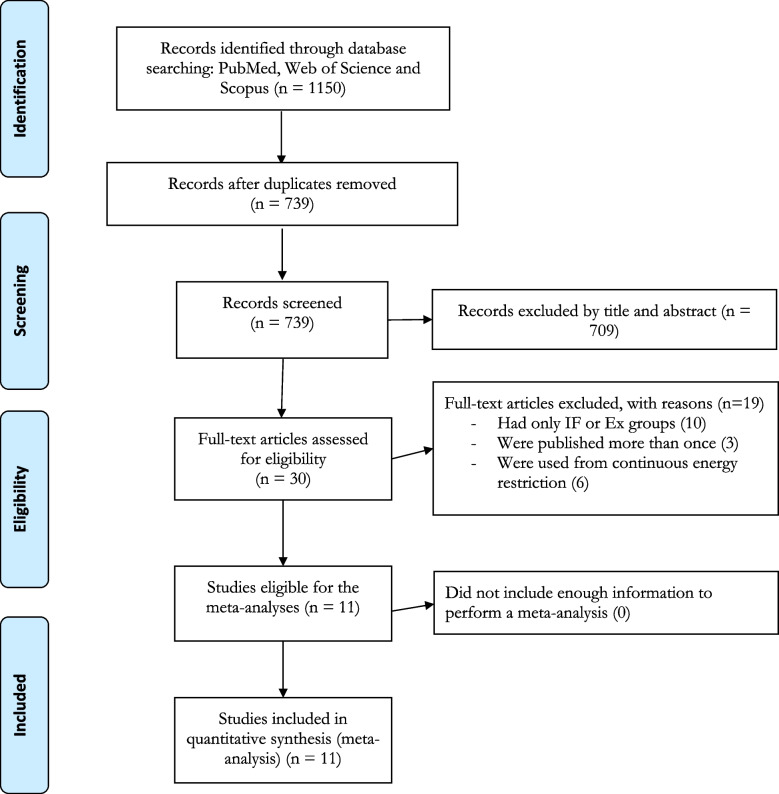
Flow diagram of systematic literature search

The search strategy yielded 267 records from PubMed, 409 records from Web of Science, and 474 records from Scopus. Following removal of duplicate records, a total of 739 records remained. After title and abstract screening, 709 additional studies were excluded. Of the remaining 30 studies, 19 studies were excluded due to the reasons presented in Fig. 1 Finally, 11 studies met all inclusion criteria, and were included in the meta-analysis. Among the 11 randomized clinical trials included, seven studies included combined EX and IF as well as Ex only, and IF only groups [37–43]; and four studies included combined EX and IF as well as Ex only groups [35, 44–46].

### Participant characteristics

A total 606 participants who were overweight or obese or had hidden obesity with mean BMI ranges from 22 to 37 kg/m^2^, and ages ranging from 21 to 45 years, were included in the meta-analysis. Sample sizes of individual studies ranged from 20 to 98. Among those studies, three included females only [37, 38, 43], one included males only [45], and seven studies included both males and females [35, 39–42, 44, 46]. One study included adults with obesity and non-alcoholic fatty liver disease [42] and one included adults with hidden obesity [37].

### Exercise and intermittent fasting characteristics

The Ex and IF characteristics are summarized in Table 1. Briefly, all included studies used supervised exercise sessions with intervention durations ranging from 4 weeks [35, 45] to 16 weeks [41], and frequency of exercise sessions ranging from 3 to 5 sessions per week. For Ex modes, four studies used HIIT [35, 38, 41, 43], three studies used aerobic training [37, 39, 42], and four studies used combined training including resistance and aerobic or interval training [40, 44–46]. For IF, seven studies used 5:2 or 4:3 feeding and fasting days [35, 38–42, 46], three studies used TRE protocols [37, 43, 44], and one study used RIF [45].

**Table 1:** Participants and intervention characteristics

Source, year	Study characteristics				Participant characteristics			Exercise intervention characteristics			Dietary intervention characteristics
	Sample size (sex)	Intervention Groups	Intervention duration (~ week)	Outcomes	Health status	Age (years)	BMI (kg/m^2^)	Type	Protocol	Weekly sessions (weekly min)	
Batitucci, et al. 2022 [38]	60 (F)	EX DI EX + DI	8 weeks	Weight, BMI, WC, BF (%), FFM, VO_2max_	Obesity	EX:33.0 ± 3.0 DI:30.0 ± 5.0 EX + DI:32.0 ± 4.4	EX:31.8 ± 2.02 DI:34.8 ± 3.2 EX + DI:34.0 ± 2.6	HIIT	Exercise execution 30-45 s at 70–85% HR_max_ with 15-30 s recovery, total 25 min duration	3 Supervised sessions (75)	IF protocol: 5:2 (5 days’ ad libitum & 2 days fasting); 6:18 h (6 h eating period & 18 h of complete fasting); 25% of the total daily energy needs on IF days (approximate total of 600 kcal that were divided into two meals), while on the other days of the week food was allowed ad libitum
Bhutani et al. 2013c [39]	67 (M & F)	EX DI EX + DI	12 weeks	Weight, BMI, BF (kg), FFM, WC, Chol, TG, HDL, LDL, Glucose, Insulin, SBP, DBP	Obesity	EX:42.0 ± 9.8 DI:42.0 ± 10.0 EX + DI:45.0 ± 21.1	EX:35.0 ± 4.9 DI:35.0 ± 5.0 EX + DI:35.0 ± 4.2	Aerobic	25–40 min at 60–75% of HR_max_	3 supervised sessions (75–120)	IF protocol: Week 1–4: Consumed 25% of their baseline energy needs on the fast day (24 h) and consumed food ad libitum on each feed day (24 h) Week 5–12: continued with the IF regimen but no fast day food was provided to them, fast day meals were consumed between 12.00 pm and 2.00 pm to ensure that each subject was undergoing the same duration of fasting, 30% kcal from fat, 15% kcal from protein, and 55% kcal from carbohydrate
Cho et al. 2019 [40]	78 (M & F)	EX DI EX + DI	8 weeks	Weight, BMI, BF (kg), Visceral fat, Glucose, Insulin, Chol, TG, HDL, LDL, VO_2max_	Overweight or obesity	EX:38.6 ± 8.2 DI:33.5 ± 5.0 EX + DI:34.5 ± 5.7	EX:26.9 ± 3.9 DI:27.8 ± 3.4 EX + DI:28.0 ± 2.6	Combined	40 min resistance exercise including weight training machines, barbells, and dumbbells; and 20 min aerobic exercise	3 supervised sessions (180)	IF protocol: 3:4 (3 days fasting & 4 days’ ad libitum), 25% of their daily recommended energy intake (approximately 500 kcal) on each “fast day” (24 h), and consumed food ad libitum on each “feed day” (24 h)
Cooke et al. 2022 [41]	45 (M & F)	EX DI EX + DI	16 weeks	Weight, Visceral fat, BF (kg), FFM, WC, VO_2max_, Glucose, TG, Chol, HDL, LDL, SBP, DBP	Overweight or obesity	EX:32.0 ± 8.3 DI:37.0 ± 5.9 EX + DI:39.0 ± 6.8	EX:32.0 ± 4.4 DI:30.0 ± 3.9 EX + DI:34.0 ± 2.1	HIIT	4–6 × 20 s cycling at 150% VO_2peak_ followed by 40 s of active recovery, total 10 min duration	3 supervised sessions (30)	IF protocols: 5:2 (2 days fasting & 5 days’ ad libitum), fasting day intake was limited to a single 600 kilocalorie (kcal) foe men and 500 kcal meal for women
Ezpeleta et al. 2023 [42]	60 (M & F)	EX DI EX + DI	12 weeks	Weight, BMI, BF (kg), Visceral fat, FFM	Obesity and NAFLD	EX:44.0 ± 13.4 DI:44.0 ± 13.4 EX + DI:44.0 ± 13.4	EX:37.0 ± 26.8 DI:36.0 ± 35.8 EX + DI:37.0 ± 22.4	Aerobic	60 min at 65–80% of HR_max_	5 supervised sessions (300)	IF protocol: 600 kcal/2,500 kJ “fast day” alternated with an ad libitum intake “feast day”. The feast and fast days began at 12 am each day. Therefore, subjects fasted for approximately 17–20 h on the fast day
Haganes et al. 2022 [43]	98 (F)	EX DI EX + DI	7 weeks	Glucose, Insulin, Weight, BF (kg), Visceral fat, VO_2max_, TG, Chol, HDL, LDL, SBP, DBP	Overweight or obesity	EX:34.9 ± 7.0 DI:36.2 ± 5.9 EX + DI:37.3 ± 5.7	EX:32.5 ± 4.5 DI:31.8 ± 3.3 EX + DI:31.4 ± 4.0	HIIT	4 × 4-min work bouts at 90–95% HR_max_, separated by 3 min moderate-intensity recovery, while the third session comprised 10 × 1-min work bouts at ≥ 90% of HR_max_ separated by 1 min low-intensity recovery	3 supervised sessions (108)	TRE protocol: ≤ 10-h daily eating window, with ad libitum energy intake
Hottenrott et al. 2020 [46]	40 (M & F)	EX EX + DI	12 weeks	Weight, BF (kg), Visceral fat	Overweight	EX:45.5 ± 7.8 EX + DI:45.5 ± 7.8	25–30 kg/m^2^	Combined	30–60 min running and 20 min of strength training (training plans based on performance status and individual heart rate training zones)	3–4 supervised sessions (ND)	IF protocol: 4:3 (3 days fasting & 4 days’ ad libitum) for the first 3 weeks, IF started with half-day fasting (800/1200 kcal per day, women/men), After 3 weeks: IF program: 5:2 (2 days fasting & 5 days’ ad libitum) with a daily caloric intake of 400 kcal for female and 600 kcal for male
Kotarsky et al. 2021 [44]	23 (M & F)	EX EX + DI	8 weeks	Weight, BMI, WC, Visceral fat, BF (kg), FFM, Insulin, Chol, HDL, SBP, DBP	Overweight and obesity	EX:44.0 ± 6.3 EX + DI:45.0 ± 9.9	EX:29.4 ± 2.5 EX + DI:29.8 ± 2.7	Combined	75 min aerobic exercise at moderate-to-vigorous (≥ 55% HRR) intensity, and resistance exercises, 3 sets with 12 reps	3 supervised sessions (~ 150–300)	TRE protocol: consume all their calories between 12:00 p.m. and 8:00 p.m. each day, inducing a fasting window of 16 h
Maaloul et al. 2023 [45]	20 (M)	DI EX + DI	4 weeks	Weight, BF (kg), Glucose, Chol, TG, HDL, LDL	Obesity	DI:33.4 ± 7.9 EX + DI:30.2 ± 6.1	DI:32.06 ± 3.6 EX + DI:34.1 ± 4.9	Combined	8 intervals of 1 min at 90% of VO_2max_ alternated with 2 min recovery at 45% of VO_2max_, and resistance exercised; 3 sets with 10–12 reps at 60–75% of 1RM	4 supervised sessions (ND)	Ramadan diurnal intermittent fasting protocol: the daily fasting duration during this study was approximately 16 h
Liu et al. 2023 [37]	58 (F)	EX DI EX + DI	8 weeks	Weight, BMI, BF, Chol, TG, HDL, LDL, SBP, DBP, LBM	Hidden obesity	EX:20.1 ± 1.4 DI:20.3 ± 1.8 EX + DI:19.9 ± 0.6	EX:21.4 ± 1.6 DI:21.6 ± 1.2 EX + DI:21.6 ± 1.5	Aerobic	11,000–12,499 steps per day	7 unsupervised sessions (ND)	Time-restricted feeding protocol: time-restricted feeding for 8 h, that is, eating at their discretion between 10:00 and 18:00 daily and fasting for the remainder of the day
Xu et al. 2022 [35]	57 (M & F)	EX EX + DI	4 weeks	Weight, BMI, LBM, FM, WC, TG, Chol, HDL, LDL, VO_2max_	Overweight or obesity	21.3 ± 2.24	EX:26.1 ± 2.0 EX + DI:26.9 ± 1.5	HIIT	5 intervals of 3 min at 80% of VO_2max_ separated by brief periods at 50% (Total time:30 min)	5 supervised sessions (150)	IF protocol: Intermittent energy restriction protocol: 2:5, 30% of their daily recommended energy intake (approximately 500–1,000 kcal)

### Meta-analysis

#### Body composition

##### Combined Ex and IF vs. Ex

Combined Ex and IF decreased body weight [WMD: -3.03 kg (95% CI: -3.44 to -2.61), *p* = 0.001; 10 trials], BMI [WMD: -1.12 kg.m^2^ (95% CI: -1.28 to -0.95), *p* = 0.001; 7 trials], body fat [SMD: -0.72 (95% CI: -1.23 to -0.21), *p* = 0.005; 10 trials], visceral fat [SMD: -0.34 (95% CI: -0.63 to -0.05), *p* = 0.01; 6 trials], and waist circumference [WMD: -2.63 cm (95% CI: -4.16 to -1.11), *p* = 0.001; 5 trials], but did not change LBM [SMD: -0.04 (95% CI: -0.35 to 0.25), *p* = 0.76; 6 trials] significantly more than Ex alone (Supplementary Figs. 1–6). Heterogeneity was not significant for body weight (I^2^ = 0.00, *p* = 0.69), BMI (I^2^ = 0.00, *p* = 0.56), visceral fat mass (I^2^ = 0.00, *p* = 0.65), and LBM (I^2^ = 8.32, *p* = 0.36), but there was high heterogeneity for body fat (I^2^ = 78.64, *p* = 0.001) and waist circumference (I^2^ = 78.25, *p* = 0.001). Visual interpretation of funnel plots suggested publication bias, but Egger’s tests only confirmed bias for body fat (*p* = 0.009), not for body weight (*p* = 0.46), BMI (*p* = 0.15), waist circumference (*p* = 0.64), or LBM (*p* = 0.15). In addition, neither visual interpretation of funnel plots or Egger’s tests showed publication bias for visceral fat mass (*p* = 0.25) (Supplementary Figs. 31–36). Sensitivity analyses for body weight and body fat showed that significance did not change by removing any individual study, but high heterogeneity for body fat may be explained by the study of Xu et al. 2022 [35] (see Supplementary Table 2). In addition, when the missing studies were accounted using the trim and fill method, the overall changes were as follow: body weight [WMD: -3.15 kg (95% CI: -3.66 to -2.63)], BMI [WMD: -1.16 kg.m^2^ (95% CI: -1.37 to -0.96)], body fat [SMD: -1.05 (95% CI: -1.58 to -0.51)], waist circumference [WMD: -2.50 cm (95% CI: -4.10 to -0.89)] and LBM [SMD: -0.09 (95% CI: -0.37 to 0.18)].

##### Combined Ex and IF vs. IF

Combined Ex and IF did not decrease body weight [WMD: -0.13 kg (95% CI: -1.46 to 1.18), *p* = 0.83; 8 trials], BMI [WMD: -0.06 kg.m^2^ (95% CI: -0.65 to 0.52), *p* = 0.83; 5 trials], body fat [SMD: 0.02 (95% CI: -0.26 to 0.31), *p* = 0.86; 8 trials], visceral fat mass [SMD: -0.16 (95% CI: -0.53 to 0.21), *p* = 0.39; 4 trials], or waist circumference [WMD: -3.42 cm (95% CI: -6.90 to 0.04), *p* = 0.05; 3 trials], or affect LBM [SMD: 0.08 (95% CI: -0.26 to 0.43), *p* = 0.62; 4 trials], significantly more compared with IF alone (Supplementary Figs. 7–12). The heterogeneity was not significant among for body weight (I^2^ = 0.00, *p* = 0.99), BMI (I^2^ = 0.00, *p* = 0.96), body fat (I^2^ = 23.18, *p* = 0.24), visceral fat (I^2^ = 13.22, *p* = 0.32), LBM (I^2^ = 0.00, *p* = 0.58), or waist circumference (I^2^ = 0.00, *p* = 0.83). Visual interpretation of funnel plots suggested publication bias for all outcomes except waist circumference, and Egger’s tests indicated bias for body weight (*p* = 0.06) and waist circumference (*p* = 0.05), but not for BMI (*p* = 0.39), body fat (*p* = 0.89), visceral fat mass (*p* = 0.43), or LBM (*p* = 0.66) (Supplementary Figs. 37–42). In addition, when the missing studies were accounted using the trim and fill method, the overall changes were as follow: body weight [WMD: 0.11 kg (95% CI: -1.08 to 1.31)], BMI [WMD: -0.00 kg.m^2^ (95% CI: -0.57 to 0.55)], body fat [SMD: 0.13 (95% CI: -0.13 to 0.39)], visceral fat mass [SMD: -0.23 (95% CI: -0.58 to 0.10)] and LBM [SMD: 0.02 (95% CI: -0.29 to 0.34)].

#### Lipid profiles

##### Combined Ex and IF vs. Ex

Combined Ex and IF did not decrease TG [WMD: 3.18 mg/dl (95% CI: -7.77 to 14.14), *p* = 0.56; 5 trials], TC [WMD: 3.77 mg/dl (95% CI: -4.43 to 11.98), *p* = 0.36; 6 trials], or LDL [WMD: -2.18 mg/dl (95% CI: -10.78 to 6.42), *p* = 0.61; 6 trials]; and did not increase HDL [WMD: 0.11 mg/dl (95% CI: -4.53 to 4.77), *p* = 0.96; 6 trials] compared with Ex alone (Supplementary Figs. 13–16). Heterogeneity was not significant for TG (I^2^ = 0.00, *p* = 0.63), TC (I^2^ = 0.00, *p* = 0.85) or LDL (I^2^ = 0.00, *p* = 0.55), but there were moderate and non-significant heterogeneity for HDL (I^2^ = 50.04, *p* = 0.07). Visual interpretation of funnel plots suggested publication bias for TC, TG, and LDL, but Egger’s tests indicated bias for TC (*p* = 0.05), and not for TG (*p* = 0.85), LDL (*p* = 0.40), or HDL (*p* = 0.74) (Supplementary Figs. 43–46). In addition, when the missing studies were accounted for using the trim and fill method, the overall changes were as follow: TG [WMD: 2.50 mg/dl (95% CI: -8.36 to 13.38)], TC [WMD: 5.87 mg/dl (95% CI: -1.68 to 13.44)] and LDL [WMD: 0.72 mg/dl (95% CI: -8.25 to 9.71].

##### Combined Ex and IF vs. IF

Combined Ex and IF did not decrease TG [WMD: -6.93 mg/dl (95% CI: -26.55 to 12.67), *p* = 0.48; 6 trials], TC [WMD: -4.40 mg/dl (95% CI: -12.79 to 3.98), *p* = 0.30; 6 trials], or LDL [WMD: -8.85 mg/dl (95% CI: -20.76 to 3.06), *p* = 0.14; 6 trials]; and did not increase HDL [WMD: 1.34 mg/dl (95% CI: -2.26 to 4.95), *p* = 0.46; 6 trials] significantly as compared with IF alone (Supplementary Figs. 17–20). Heterogeneity was not significant for TC (I^2^ = 0.00, *p* = 0.76), but there were small and non-significant heterogeneity for LDL (I^2^ = 46.71, *p* = 0.09) and HDL (I^2^ = 26.00, *p* = 0.23) and moderate and significant heterogeneity for TG (I^2^ = 55.65, *p* = 0.04). Visual interpretation of funnel plots suggested publication bias, but Egger’s tests did not confirm for bias for TG (*p* = 0.21), TC (*p* = 0.61), LDL (*p* = 0.65), or HDL (*p* = 0.38) (Supplementary Figs. 47–50). In addition, when the missing studies were accounted using the trim and fill method, the overall changes were as follow**:** TG [WMD: 2.09 mg/dl (95% CI: -19.78 to 23.96)], TC [WMD: -3.36 mg/dl (95% CI: -11.43 to 4.69)], LDL [WMD: -14.37 mg/dl (95% CI: -27.00 to -1.74)] and HDL [WMD: 0.14 mg/dl (95% CI: -3.94 to 4.24)].

#### Blood pressure

##### Combined Ex and IF vs. Ex

Combined Ex and IF did not decrease SBP [WMD: -1.70 mmHg (95% CI: -4.57 to 1.16), *p* = 0.24; 6 trials] or DBP [WMD: -0.21 mmHg (95% CI: -2.51 to 2.09), *p* = 0.85; 6 trials] as compared with IF alone (Supplementary Figs. 21 and 22). The heterogeneity was not significant for SBP (I^2^ = 0.00, *p* = 0.68) or DBP (I^2^ = 0.00, *p* = 0.50). Visual interpretation of funnel plots suggested publication bias for SBP, but Egger’s tests did not confirm bias for SBP (*p* = 0.57) or DBP (*p* = 0.48) (Supplementary Figs. 51 and 52). In addition, when the missing studies were accounted using the trim and fill method, the overall changes were as follow**:** SBP [WMD: -1.35 mmHg (95% CI: -4.05 to 1.33)] and DBP [WMD: 0.65 mmHg (95% CI: -1.39 to 2.69)].

##### Combined Ex and IF vs. IF

Combined Ex and IF did not decrease SBP [WMD: -3.00 mmHg (95% CI: -6.06 to 0.05), *p* = 0.05; 5 trials] or DBP [WMD: -0.11 mmHg (95% CI: -2.73 to 2.51), *p* = 0.93; 5 trials] significantly as compared with IF alone (Supplementary Figs. 23 and 24). The heterogeneity was not significant for SBP (I^2^ = 0.00, *p* = 0.63) or DBP (I^2^ = 0.00, *p* = 0.86). Visual interpretation of funnel plots suggested publication bias for SBP, but Egger’s tests did not indicate bias for SBP (*p* = 0.12) or DBP (*p* = 0.94) (Supplementary Figs. 53 and 54). In addition, when the missing studies were accounted using the trim and fill method, the overall changes were as follow**:** SBP [WMD: -3.85 mmHg (95% CI: -6.66 to 1.01)] or DBP [WMD: 0.58 mmHg (95% CI: -1.72 to 2.89)].

#### Glycemia

##### Combined Ex and IF vs. Ex

Combined Ex and IF did not significantly decrease glucose [WMD: -1.89 mg/dl (95% CI: -6.18 to 2.39), *p* = 0.38; 4 trials] or insulin [SMD: -0.24 (95% CI: -0.58 to 0.10), *p* = 0.16; 4 trials] compared with IF alone (Supplementary Figs. 25 and 26). Heterogeneity was not significant for insulin (I^2^ = 0.00, *p* = 0.65), but there was small and non-significant heterogeneity for glucose (I^2^ = 37.86, *p* = 0.18). Visual interpretation of funnel plots suggested publication bias for glucose, but Egger’s tests did not confirm this bias for glucose (*p* = 0.25) or insulin (*p* = 0.85) (Supplementary Figs. 55 and 56). In addition, when the missing studies were accounted using the trim and fill method, the overall change was as follow**:** glucose [WMD: -2.42 mg/dl (95% CI: -6.16 to 1.31)].

##### Combined Ex and IF vs. IF

Combined Ex and IF did not significantly decrease glucose [WMD: -1.81 mg/dl (95% CI: -4.93 to 1.29), *p* = 0.25; 5 trials] or insulin [SMD: -0.24 (95% CI: -0.62 to 0.13), *p* = 0.20; 3 trials] compared with IF alone (Supplementary Figs. 27 and 28). The heterogeneity was not significant for glucose (I^2^ = 0.00, *p* = 0.55) or insulin (I^2^ = 0.00, *p* = 0.41). Visual interpretation of funnel plots and the Egger’s test did not show publication bias for glucose (*p* = 0.48). However, visual interpretation of funnel plots suggested publication bias for insulin, whereas the Egger’s test did not (*p* = 0.36) (Supplementary Figs. 57 and 58). In addition, when the missing studies were accounted using the trim and fill method, the overall change was as follow**:** insulin [SMD: -0.14 (95% CI: -0.55 to 0.26)].

#### VO_2_max/peak

##### Combined Ex and IF vs. Ex

Combined Ex and IF did not significantly increase VO_2max/peck_ [SMD: 0.26 (95% CI: -0.10 to 0.63), *p* = 0.15; 4 trials] compared with Ex alone (Supplementary Fig. 29). The heterogeneity was no significant (I^2^ = 0.00, *p* = 0.95). Both visual interpretation of funnel plots and the Egger’s test did not show publication bias (*p* = 0.21) (Supplementary Fig. 59).

##### Combined Ex and IF vs. IF

Combined Ex and IF significantly increased VO_2max/peck_ [SMD: 0.55 (95% CI: 0.14 to 0.97), *p* = 0.009; 3 trials] compared with IF alone (Supplementary Fig. 30). The heterogeneity was not significant (I^2^ = 0.00, *p* = 0.99). Both visual interpretation of funnel plots and the Egger’s test did not show publication bias (*p* = 0.79) (Supplementary Fig. 60).

### Quality assessment

The overall qualities of included studies are summarized in Supplementary Table 3 which ranged from five to seven out of a maximum nine.

## Discussion

The current systematic review and meta-analysis provides evidence that the combination of Ex and IF produces superior changes in body weight, BMI, body fat, and visceral fat when compared to Ex alone. However, combined Ex and IF was only more effective than IF alone for increasing VO_2_max/peak. In contrast to our hypothesis, that combining Ex and IF did not improve lipid profiles, glycemic outcomes, or blood pressure compared to Ex or IF alone. Overall, these findings indicated that adopting both Ex and IF may be more effective for weight and fat loss, but not for other cardiovascular risk factors.

Previous reviews and meta-analyses have confirmed that combined Ex and IF interventions are effective for decreasing body fat and visceral fat mass in overweight and obese adults [47, 48]. Ex promotes weight loss mainly through enhanced energy expenditure [49]. However, without a dietary intervention combined with Ex, it is not as effective for weight loss [50]. Dietary energy restriction, that reduces energy intake by 15 to 60%, or IF, are more effective strategies for weight loss [47, 51], with a potential downside in a reduction of fat free mass when not combined with Ex [51]. Our findings support the recommendation of combined protocols which is similar to an earlier study, despite the difference in the type of dietary interventions adopted [30, 52, 53].

Several meta-analyses have suggested that IF produces equivalent weight loss when compared to continuous energy restriction (CER) [54, 55], offering a potential option for those who struggle to consistently restrict caloric intake. Comparison between IF and CER during Ex was not possible due to the limited number of available studies, but IF may be a suitable substitute for CER. In this regard, Xu et al., showed that both IF and CER were effective for improving body composition when combined with Ex, with IF showing larger effects [35]. In the current meta-analysis, combined Ex and IF was more effective than Ex alone for reducing visceral fat and waist circumference, that also fell by -3.42 cm compared to IF alone, though the difference was not statistically significant. Visceral fat is known to be an important and independent risk factor for cardiometabolic diseases [56] and waist circumference is the best anthropometric predictor of visceral fat [57]. On other hand, our finding shows that the combination of EX and IF did not change LBM or fat-free mass versus Ex or IF alone demonstrating role of exercise in maintaining muscle mass. This is an important finding because it is well known that the loss of muscle mass during energy restriction is a negative effect of weight loss [58, 59]. In contrast, maintaining muscle mass may increase resting metabolic rate and energy production, thereby leading to better maintenance of weight loss and potentially greater fat loss [39].

Previous meta-analyses suggest that Ex and IF intervention positively alters lipid profiles, glycemic markers, and blood pressure [16, 60–64]. However, no published meta-analysis has investigated the combination of Ex and IF versus standalone Ex and IF interventions. In contrast to our hypothesis, combined Ex and IF was not associated with significantly greater improvements in cardiometabolic health markers which contrasts with earlier meta-analyses reporting that combining Ex and dietary interventions (DI) may be more effective for improving glycemic markers and lipid profiles [28, 30]. One possible explanation for similar cardiometabolic health benefits could be the effectiveness of either standalone interventions, and there are ceiling effects regarding the magnitude of improvement. In addition, most of the included participants, despite having overweight or obesity, did not have metabolic disorders and were within a healthy range at baseline, or reached a healthy range after intervention. Therefore, the combination of interventions may be more effective in people with cardiometabolic disorders, such as those with type 2 diabetes mellitus, for improving glycemic markers; or with hypertension, for improving blood pressure, or people with dyslipidemia for improving lipid profiles.

The current meta-analysis also indicated that Ex is necessary for improving VO_2max/peak_, where combining Ex and IF is more effective than IF alone, with a moderate effect size, but not compared with Ex alone. Ex is established to be a primary intervention for improving cardiorespiratory fitness, which has been confirmed in several meta-analyses of different populations and different types of Ex [60, 65–69], even during CER [70]. Cardiorespiratory fitness is an independent cardiometabolic health marker [71, 72] which decreases during CER [73]. Therefore, our results regarding promoting cardiorespiratory fitness during weight loss suggest that IF alone is not sufficient.

The current systematic review and meta-analysis had several limitations that should be considered when interpreting the results. There were only a small numbers of studies that met the inclusion criteria with blood markers and blood pressure outcomes available. Therefore, we were not able to compared the effects of Ex or IF type. Significant heterogeneity for some outcomes was found, which may be due to the variable types of Ex and IF, that we were unable to investigate further. In addition, a majority of included studies used a short intervention period (< 12 weeks) with further studies needed to detriment potential role of combined Ex plus IF. Finally, according to the available studies, the present meta-analysis was limited to young adults and further studies are needed to determine the importance of age.

In conclusion, the current systematic review and meta-analysis provides evidence that the combination of Ex and IF is effective for promoting weight and fat loss, as well as for maintaining muscle mass and increasing cardiorespiratory fitness. However, a combination of Ex and IF is not associated with greater improvements in lipid profiles, glycemic markers, or blood pressure. Further randomized trials are required to elucidate the types of Ex and IF that when combined, have the greatest benefit for cardiometabolic health.

### Supplementary Information

**Additional file 1: Supplementary Table 1.** Search strategy. **Supplementary Table 2.** Risk of bias assessment. **Supplementary Table 3.** Sensitivity analyses. **Supplementary Figure 1.** Forest plot of the effects of Combined Ex and IF versus Ex alone on Body weight. Data are reported as WMD (95% confidence limits). WMD: weighted mean difference. **Supplementary Figure 2.** Forest plot of the effects of Combined Ex and IF versus Ex alone on BMI. Data are reported as WMD (95% confidence limits). WMD: weighted mean difference. **Supplementary Figure 3.** Forest plot of the effects of Combined Ex and IF versus Ex alone on Body fat. Data are reported as SMD (95% confidence limits). SMD: standardized mean difference. **Supplementary Figure 4.** Forest plot of the effects of Combined Ex and IF versus Ex alone on Visceral fat. Data are reported as SMD (95% confidence limits). SMD: standardized mean difference. **Supplementary Figure 5.** Forest plot of the effects of Combined Ex and IF versus Ex alone on waist circumference. Data are reported as WMD (95% confidence limits). SMD: weighted mean difference. **Supplementary Figure 6.** Forest plot of the effects of Combined Ex and IF versus Ex alone on LBM. Data are reported as SMD (95% confidence limits). SMD: standardized mean difference. **Supplementary Figure 7.** Forest plot of the effects of Combined Ex and IF versus IF alone on Body weight. Data are reported as WMD (95% confidence limits). WMD: weighted mean difference. **Supplementary Figure 8.** Forest plot of the effects of Combined Ex and IF versus IF alone on BMI. Data are reported as WMD (95% confidence limits). WMD: weighted mean difference. **Supplementary Figure 9.** Forest plot of the effects of Combined Ex and IF versus IF alone on Body fat. Data are reported as SMD (95% confidence limits). SMD: standardized mean difference. **Supplementary Figure 10.** Forest plot of the effects of Combined Ex and IF versus IF alone on Visceral fat. Data are reported as SMD (95% confidence limits). SMD: standardized mean difference. **Supplementary Figure 11.** Forest plot of the effects of Combined Ex and IF versus IF alone on Waist circumference. Data are reported as WMD (95% confidence limits). WMD: weighted mean difference. **Supplementary Figure 12.** Forest plot of the effects of Combined Ex and IF versus IF alone on LBM. Data are reported as SMD (95% confidence limits). SMD: standardized mean difference. **Supplementary Figure 13.** Forest plot of the effects of Combined Ex and IF versus Ex alone on TG. Data are reported as WMD (95% confidence limits). WMD: weighted mean difference. **Supplementary Figure 14.** Forest plot of the effects of Combined Ex and IF versus Ex alone on TC. Data are reported as WMD (95% confidence limits). WMD: weighted mean difference. **Supplementary Figure 15.** Forest plot of the effects of Combined Ex and IF versus Ex alone on LDL. Data are reported as WMD (95% confidence limits). WMD: weighted mean difference. **Supplementary Figure 16.** Forest plot of the effects of Combined Ex and IF versus Ex alone on HDL. Data are reported as WMD (95% confidence limits). WMD: weighted mean difference. **Supplementary Figure 17.** Forest plot of the effects of Combined Ex and IF versus IF alone on TG. Data are reported as WMD (95% confidence limits). WMD: weighted mean difference. **Supplementary Figure 18.** Forest plot of the effects of Combined Ex and IF versus IF alone on TC. Data are reported as WMD (95% confidence limits). WMD: weighted mean difference. **Supplementary Figure 19.** Forest plot of the effects of Combined Ex and IF versus IF alone on LDL. Data are reported as WMD (95% confidence limits). WMD: weighted mean difference. **Supplementary Figure 20.** Forest plot of the effects of Combined Ex and IF versus IF alone on HDL. Data are reported as WMD (95% confidence limits). WMD: weighted mean difference. **Supplementary Figure 21.** Forest plot of the effects of Combined Ex and IF versus Ex alone on SBP. Data are reported as WMD (95% confidence limits). WMD: weighted mean difference. **Supplementary Figure 22.** Forest plot of the effects of Combined Ex and IF versus Ex alone on DBP. Data are reported as WMD (95% confidence limits). WMD: weighted mean difference. **Supplementary Figure 23.** Forest plot of the effects of Combined Ex and IF versus IF alone on SBP. Data are reported as WMD (95% confidence limits). WMD: weighted mean difference. **Supplementary Figure 24.** Forest plot of the effects of Combined Ex and IF versus IF alone on DBP. Data are reported as WMD (95% confidence limits). WMD: weighted mean difference. **Supplementary Figure 25.** Forest plot of the effects of Combined Ex and IF versus Ex alone on Glucose. Data are reported as WMD (95% confidence limits). WMD: weighted mean difference. **Supplementary Figure 26.** Forest plot of the effects of Combined Ex and IF versus Ex alone on Insulin. Data are reported as SMD (95% confidence limits). SMD: Standardized mean difference. **Supplementary Figure 27.** Forest plot of the effects of Combined Ex and IF versus IF alone on Glucose. Data are reported as WMD (95% confidence limits). WMD: weighted mean difference. **Supplementary Figure 28.** Forest plot of the effects of Combined Ex and IF versus IF alone on Insulin. Data are reported as SMD (95% confidence limits). SMD: Standardized mean difference. **Supplementary Figure 29.** Forest plot of the effects of Combined Ex and IF versus Ex alone on VO2max/peck. Data are reported as SMD (95% confidence limits). SMD: Standardized mean difference. **Supplementary Figure 30.** Forest plot of the effects of Combined Ex and IF versus IF alone on VO2max/peck. Data are reported as SMD (95% confidence limits). SMD: Standardized mean difference. **Supplementary Figure 31.** Funnel plot of the effects of Combined Ex and IF versus Ex alone on Body weight. **Supplementary Figure 32.** Funnel plot of the effects of Combined Ex and IF versus Ex alone on BMI. **Supplementary Figure 33.** Funnel plot of the effects of Combined Ex and IF versus Ex alone on Body fat. **Supplementary Figure 34.** Funnel plot of the effects of Combined Ex and IF versus Ex alone on Visceral fat. **Supplementary Figure 35.** Funnel plot of the effects of Combined Ex and IF versus Ex alone on waist circumference. **Supplementary Figure 36.** Funnel plot of the effects of Combined Ex and IF versus Ex alone on LBM. **Supplementary Figure 37.** Funnel plot of the effects of Combined Ex and IF versus IF alone on Body weight. **Supplementary Figure 38.** Funnel plot of the effects of Combined Ex and IF versus IF alone on BMI. **Supplementary Figure 39.** Funnel plot of the effects of Combined Ex and IF versus IF alone on Body fat. **Supplementary Figure 40.** Funnel plot of the effects of Combined Ex and IF versus IF alone on Visceral fat. **Supplementary Figure 41.** Funnel plot of the effects of Combined Ex and IF versus IF alone on Waist circumference. **Supplementary Figure 42.** Funnel plot of the effects of Combined Ex and IF versus IF alone on LBM. **Supplementary Figure 43.** Funnel plot of the effects of Combined Ex and IF versus Ex alone on TG. **Supplementary Figure 44.** Funnel plot of the effects of Combined Ex and IF versus Ex alone on TC. **Supplementary Figure 45.** Funnel plot of the effects of Combined Ex and IF versus Ex alone on LDL. **Supplementary Figure 46.** Funnel plot of the effects of Combined Ex and IF versus Ex alone on HDL. **Supplementary Figure 47.** Funnel plot of the effects of Combined Ex and IF versus IF alone on TG. **Supplementary Figure 48.** Funnel plot of the effects of Combined Ex and IF versus IF alone on TC. **Supplementary Figure 49.** Funnel plot of the effects of Combined Ex and IF versus IF alone on LDL. **Supplementary Figure 50.** Funnel plot of the effects of Combined Ex and IF versus IF alone on HDL. **Supplementary Figure 51.** Funnel plot of the effects of Combined Ex and IF versus Ex alone on SBP. **Supplementary Figure 52.** Funnel plot of the effects of Combined Ex and IF versus Ex alone on DBP. **Supplementary Figure 53.** Funnel plot of the effects of Combined Ex and IF versus IF alone on SBP. **Supplementary Figure 54.** Funnel plot of the effects of Combined Ex and IF versus IF alone on DBP. **Supplementary Figure 55.** Funnel plot of the effects of Combined Ex and IF versus Ex alone on Glucose. **Supplementary Figure 56.** Funnel plot of the effects of Combined Ex and IF versus Ex alone on Insulin. **Supplementary Figure 57.** Funnel plot of the effects of Combined Ex and IF versus IF alone on Glucose. **Supplementary Figure 58.** Funnel plot of the effects of Combined Ex and IF versus IF alone on Insulin. **Supplementary Figure 59.** Funnel plot of the effects of Combined Ex and IF versus Ex alone on VO2max/peck. **Supplementary Figure 60.** Funnel plot of the effects of Combined Ex and IF versus IF alone on VO2max/peck.

## Data Availability

The data that support the findings of this study are available from the corresponding author on request.
