# Supplementary material for: Combined versus independent effects of exercise training and intermittent fasting on body composition and cardiometabolic health in adults: a systematic review and meta-analysis
Source: Nutr J. 2024 Jan 6;23:7. doi: 10.1186/s12937-023-00909-x (PMC10770891; doi:10.1186/s12937-023-00909-x)
Supplement: Supplementary file 1 — Additional file 1: Supplementary Table 1. Search strategy. Supplementary Table 2. Risk of bias assessment. Supplementary Table 3. Sensitivity analyses. Supplementary Figure 1. Forest plot of the effects of Combined Ex and IF versus Ex alone on Body weight. Data are reported as WMD (95% confidence limits). WMD: weighted mean difference. Supplementary Figure 2. Forest plot of the effects of Combined Ex and IF versus Ex alone on BMI. Data are reported as WMD (95% confidence limits). WMD: weighted mean difference. Supplementary Figure 3. Forest plot of the effects of Combined Ex and IF versus Ex alone on Body fat. Data are reported as SMD (95% confidence limits). SMD: standardized mean difference. Supplementary Figure 4. Forest plot of the effects of Combined Ex and IF versus Ex alone on Visceral fat. Data are reported as SMD (95% confidence limits). SMD: standardized mean difference. Supplementary Figure 5. Forest plot of the effects of Combined Ex and IF versus Ex alone on waist circumference. Data are reported as WMD (95% confidence limits). SMD: weighted mean difference. Supplementary Figure 6. Forest plot of the effects of Combined Ex and IF versus Ex alone on LBM. Data are reported as SMD (95% confidence limits). SMD: standardized mean difference. Supplementary Figure 7. Forest plot of the effects of Combined Ex and IF versus IF alone on Body weight. Data are reported as WMD (95% confidence limits). WMD: weighted mean difference. Supplementary Figure 8. Forest plot of the effects of Combined Ex and IF versus IF alone on BMI. Data are reported as WMD (95% confidence limits). WMD: weighted mean difference. Supplementary Figure 9. Forest plot of the effects of Combined Ex and IF versus IF alone on Body fat. Data are reported as SMD (95% confidence limits). SMD: standardized mean difference. Supplementary Figure 10. Forest plot of the effects of Combined Ex and IF versus IF alone on Visceral fat. Data are reported as SMD (95% confidence limits). SMD: standardized mean [file 12937_2023_909_MOESM1_ESM.docx]

**Supplementary Table and Figures Title**

**Supplementary Table 1. Search strategy**

| Databases | Search strategy | Limits | Results |
| --- | --- | --- | --- |
| PubMed | ("time-restricted feeding"[All Fields] OR "time-restricted feeding"[All Fields] OR "time-restricted eating"[All Fields] OR "time-restricted eating"[All Fields] OR "time-restricted diet"[All Fields] OR "time-restricted diet"[All Fields] OR "time-restricted fasting"[All Fields] OR "time-restricted fasting"[All Fields] OR "intermittent fasting"[All Fields] OR "intermittent energy restriction"[All Fields] OR "alternate fasting"[All Fields] OR "periodic fasting"[All Fields] OR "reduced meal frequency"[All Fields] OR "alternate-day fasting"[All Fields]) AND ("exercise"[MeSH Terms] OR "exercise"[All Fields] OR "exercises"[All Fields] OR "exercise therapy"[MeSH Terms] OR ("exercise"[All Fields] AND "therapy"[All Fields]) OR "exercise therapy"[All Fields] OR "exercising"[All Fields] OR "exercise s"[All Fields] OR "exercised"[All Fields] OR "exerciser"[All Fields] OR "exercisers"[All Fields] OR "exercise training"[All Fields] OR "physical activity"[All Fields] OR "aerobic training"[All Fields] OR "aerobic exercise"[All Fields] OR "resistance training"[All Fields] OR "resistance exercise"[All Fields] OR "combined training"[All Fields] OR "combined exercise"[All Fields] OR "concurrent training"[All Fields] OR "concurrent exercise"[All Fields] OR "interval training"[All Fields] OR "interval exercise"[All Fields]) | Humans, English | 267 |
| Scopus | ( TITLE-ABS-KEY ( "time-restricted feeding" OR "time restricted feeding" OR "time-restricted eating" OR "time restricted eating" OR "time-restricted diet" OR "time restricted diet" OR "time-restricted fasting" OR "time restricted fasting" OR "intermittent fasting" OR "intermittent energy restriction" OR "alternate fasting" OR "periodic fasting" OR "reduced meal frequency" OR "alternate-day fasting" ) AND TITLE-ABS-KEY ( exercise OR "exercise training" OR "physical activity" OR "aerobic training" OR "aerobic exercise" OR "resistance training" OR "resistance exercise" OR "combined training" OR "combined exercise" OR "concurrent training" OR "concurrent exercise" OR "interval training" OR "interval exercise" ) ) | Journal | 474 |
| Web of science | (ALL=("time-restricted feeding" OR "time restricted feeding" OR "time-restricted eating" OR "time restricted eating" OR "time-restricted diet" OR "time restricted diet" OR "time-restricted fasting" OR "time restricted fasting" OR "intermittent fasting" OR "intermittent energy restriction" OR "alternate fasting" OR "periodic fasting" OR "reduced meal frequency" OR "alternate-day fasting")) AND ALL=( exercise OR "exercise training" OR "physical activity" OR "aerobic training" OR "aerobic exercise" OR "resistance training" OR "resistance exercise" OR "combined training" OR "combined exercise" OR "concurrent training" OR "concurrent exercise" OR "interval training" OR "interval exercise") | English | 409 |

**Supplementary Table 2.** Risk of bias assessment

| **Authors & Year** | **Criteria 1** | **Criteria 2** | **Criteria 3** | **Criteria 4** | **Criteria 5** | **Criteria 6** | **Criteria 7** | **Criteria 8** | **Criteria 9** | **total** |
| --- | --- | --- | --- | --- | --- | --- | --- | --- | --- | --- |
| Batitucci, et al. 2022 [1] | ✓ | ✓ | x | ✓ | x | ✓ | x | ✓ | ✓ | 6 |
| Bhutani et al. 2013 [2] | ✓ | ✓ | x | x | x | x | ✓ | ✓ | ✓ | 5 |
| Cho et al. 2019 [3] | ✓ | ✓ | ✓ | ✓ | ✓ | x | x | ✓ | ✓ | 7 |
| Cooke et al. 2022 [4] | ✓ | ✓ | x | ✓ | x | x | ✓ | ✓ | ✓ | 6 |
| Ezpeleta et al. 2023 [5] | ✓ | ✓ | x | ✓ | ✓ | ✓ | x | ✓ | ✓ | 7 |
| Haganes et al. 2022 [6] | ✓ | ✓ | x | ✓ | x | ✓ | ✓ | ✓ | ✓ | 7 |
| Hottenrott et al. 2020 [7] | ✓ | ✓ | ? | ✓ | ✓ | ✓ | x | ✓ | ✓ | 7 |
| Kotarsky et al. 2021 [8] | ✓ | ✓ | ? | ✓ | x | ✓ | x | ✓ | ✓ | 6 |
| Liu et al. 2023 [9] | ✓ | ✓ | x | ✓ | x | ✓ | x | ✓ | ✓ | 6 |
| Maaloul et al. 2023 [10] | ✓ | ✓ | ? | ✓ | x | ✓ | x | ✓ | ✓ | 6 |
| Moro et al. 2021 [11] | ? | ✓ | x | ? | x | ✓ | x | ✓ | ✓ | 4 |
| Oh et al. 2018 [12] | ✓ | ✓ | x | ✓ | ✓ | x | x | ✓ | ✓ | 6 |
| Xu et al. 2022 [13] | ✓ | ✓ | x | ✓ | x | x | x | ✓ | ✓ | 5 |

1. Eligibility Criteria specified, (2) Random allocation of participants, (3) Allocation concealed, (4) Groups similar at baseline, (5) Assessors blinded, (6) Outcome measures assessed in 85% of participants, (7) Intention to treat analysis, (8) Reporting of between group statistical comparison, (9) Point measures and measures of variability reported for main effects. ‘low (✓), ‘high (x) and unclear (?)

Supplementary Table 3. Sensitivity analyses

| Group | Outcomes | Range | SMD or WMD (95% CIs) | P | I^2^ |
| --- | --- | --- | --- | --- | --- |
| Combined Ex and IF vs. Ex | Body weight | Minimum | -2.36 kg (-3.40 to -1.33) | 0.001 | 0.00% |
|  |  | Maximum | -3.10 kg (-3.53 to -2.67) | 0.001 | 0.00% |
|  | Body fat | Minimum | -0.39 (-0.64 to -0.14) | 0.002 | 14.39% |
|  |  | Maximum | -0.81 (-1.37 to -0.25) | 0.005 | 79.96% |


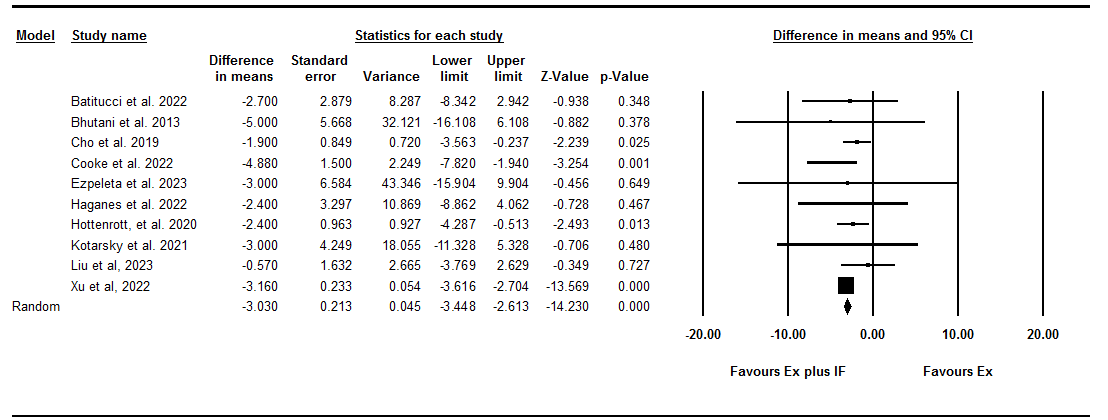
Supplementary Figure 1. Forest plot of the effects of Combined Ex and IF versus Ex alone on Body weight. Data are reported as WMD (95% confidence limits). WMD: weighted mean difference.


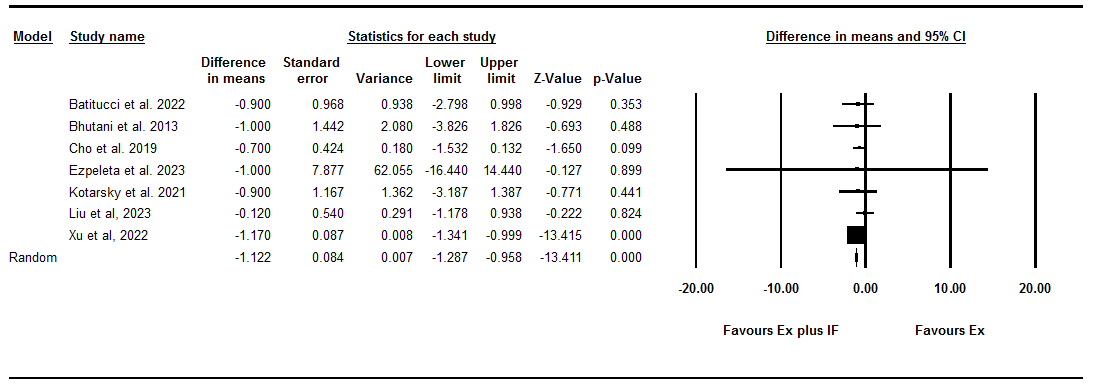
Supplementary Figure 2. Forest plot of the effects of Combined Ex and IF versus Ex alone on BMI. Data are reported as WMD (95% confidence limits). WMD: weighted mean difference.


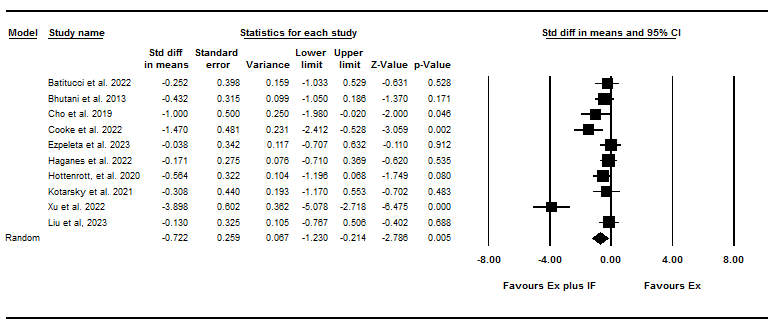
Supplementary Figure 3. Forest plot of the effects of Combined Ex and IF versus Ex alone on Body fat. Data are reported as SMD (95% confidence limits). SMD: standardized mean difference.


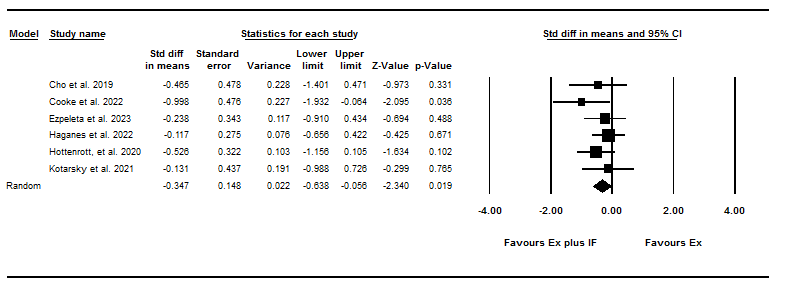
Supplementary Figure 4. Forest plot of the effects of Combined Ex and IF versus Ex alone on Visceral fat. Data are reported as SMD (95% confidence limits). SMD: standardized mean difference.


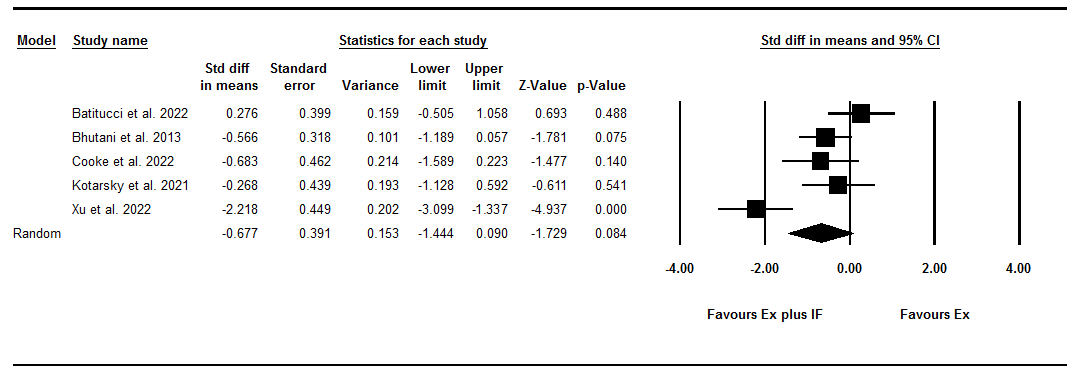
Supplementary Figure 5. Forest plot of the effects of Combined Ex and IF versus Ex alone on waist circumference. Data are reported as WMD (95% confidence limits). SMD: weighted mean difference.


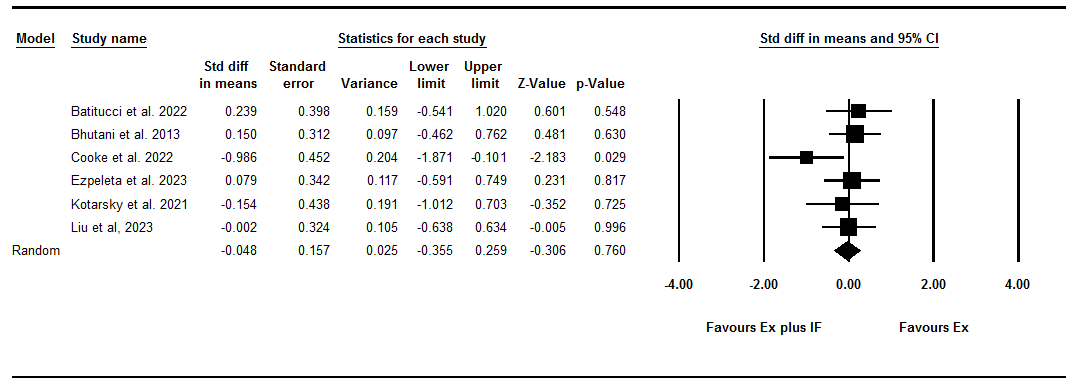
Supplementary Figure 6. Forest plot of the effects of Combined Ex and IF versus Ex alone on LBM. Data are reported as SMD (95% confidence limits). SMD: standardized mean difference.


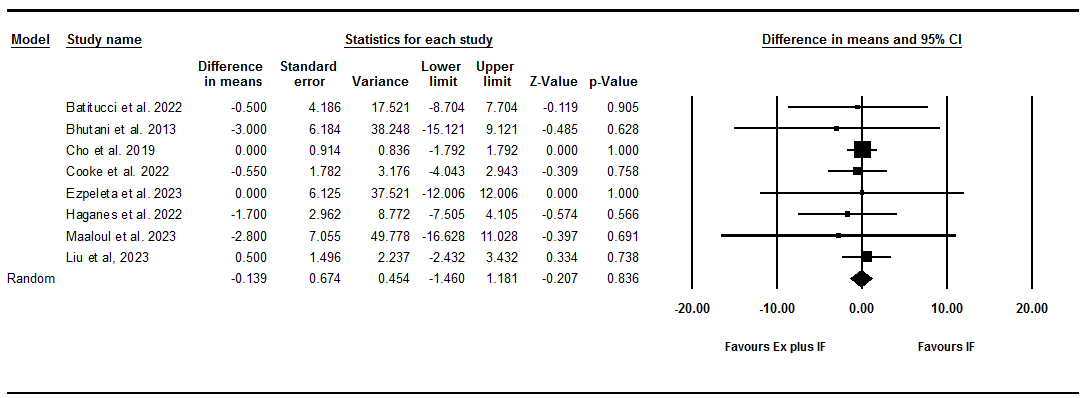
Supplementary Figure 7. Forest plot of the effects of Combined Ex and IF versus IF alone on Body weight. Data are reported as WMD (95% confidence limits). WMD: weighted mean difference.


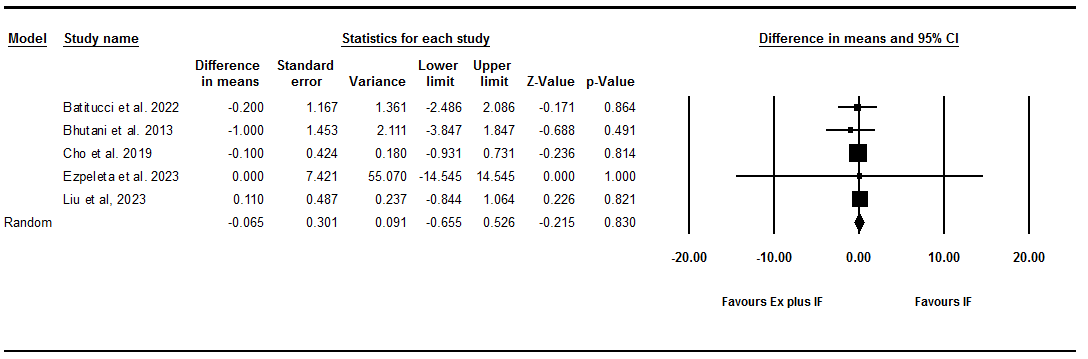
Supplementary Figure 8. Forest plot of the effects of Combined Ex and IF versus IF alone on BMI. Data are reported as WMD (95% confidence limits). WMD: weighted mean difference.


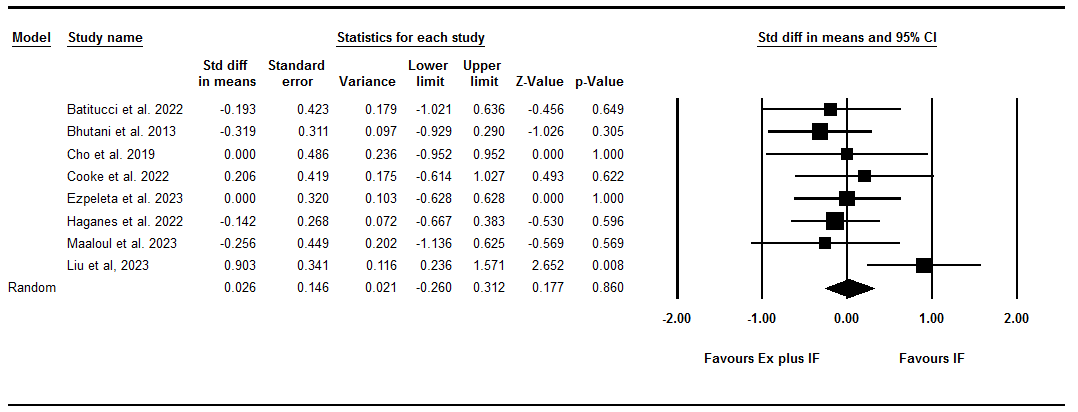
Supplementary Figure 9. Forest plot of the effects of Combined Ex and IF versus IF alone on Body fat. Data are reported as SMD (95% confidence limits). SMD: standardized mean difference.


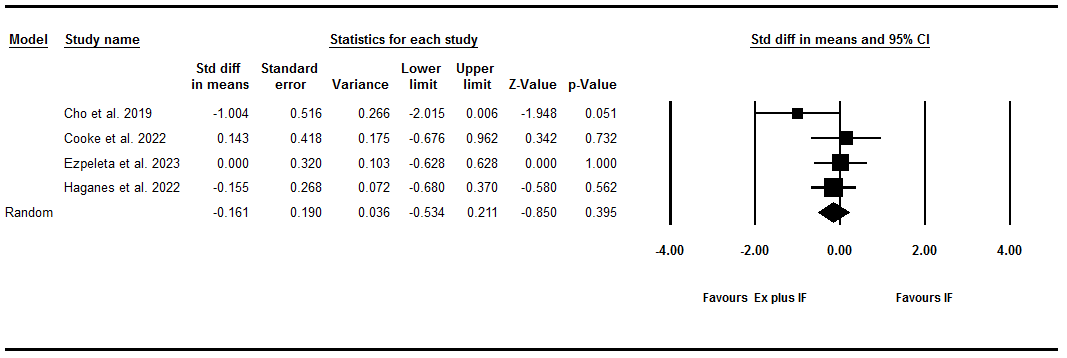
Supplementary Figure 10. Forest plot of the effects of Combined Ex and IF versus IF alone on Visceral fat. Data are reported as SMD (95% confidence limits). SMD: standardized mean difference.


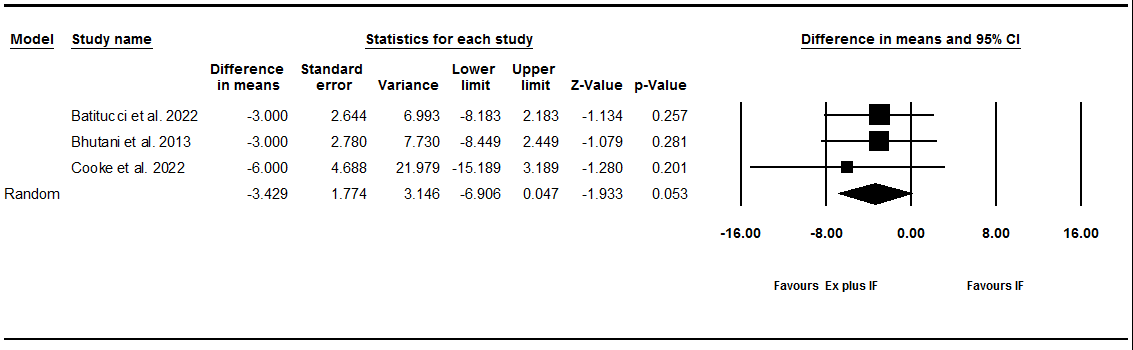
Supplementary Figure 11. Forest plot of the effects of Combined Ex and IF versus IF alone on Waist circumference. Data are reported as WMD (95% confidence limits). WMD: weighted mean difference.


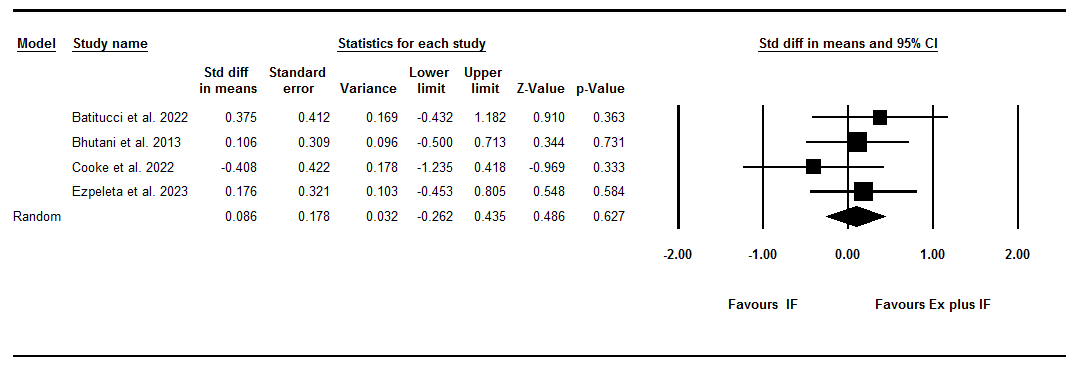
Supplementary Figure 12. Forest plot of the effects of Combined Ex and IF versus IF alone on LBM. Data are reported as SMD (95% confidence limits). SMD: standardized mean difference.


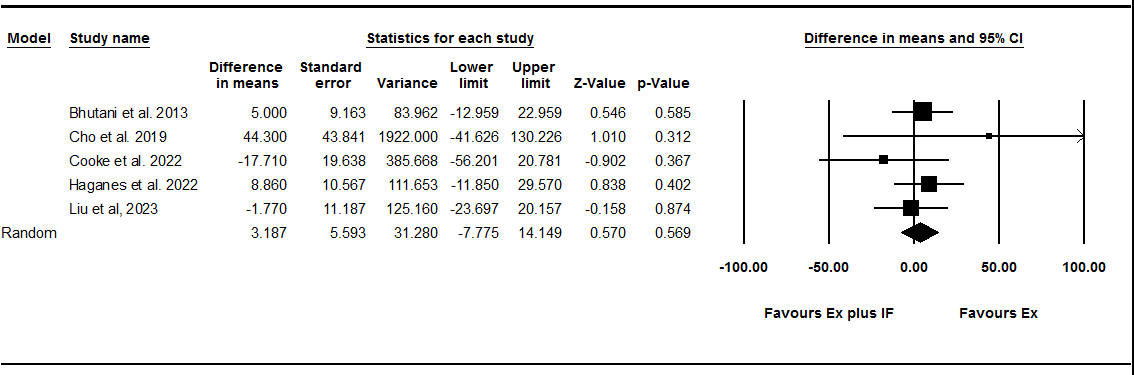
Supplementary Figure 13. Forest plot of the effects of Combined Ex and IF versus Ex alone on TG. Data are reported as WMD (95% confidence limits). WMD: weighted mean difference.


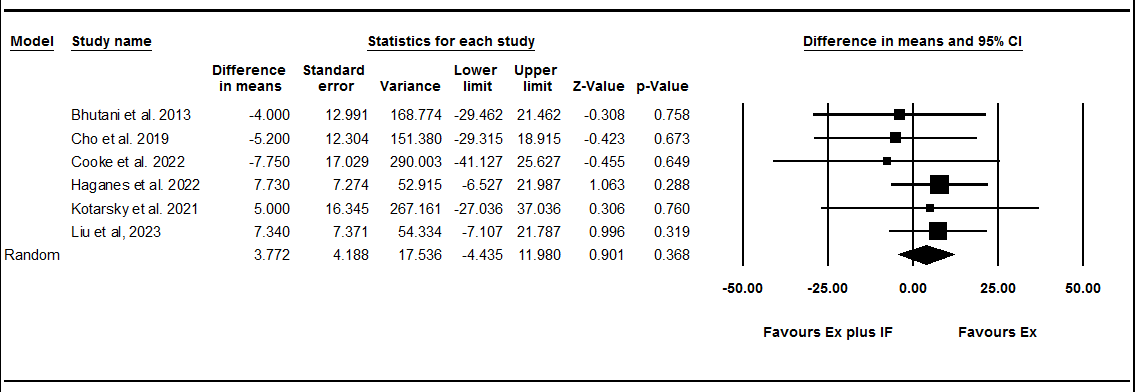
Supplementary Figure 14. Forest plot of the effects of Combined Ex and IF versus Ex alone on TC. Data are reported as WMD (95% confidence limits). WMD: weighted mean difference.


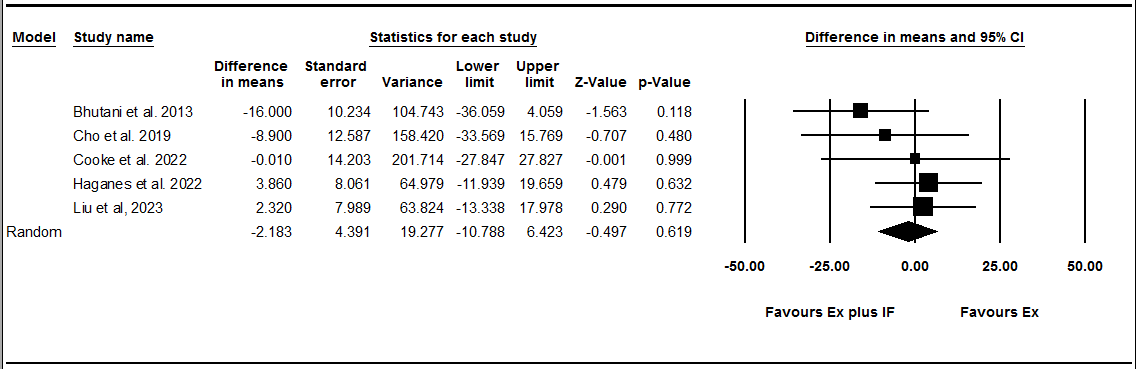
Supplementary Figure 15. Forest plot of the effects of Combined Ex and IF versus Ex alone on LDL. Data are reported as WMD (95% confidence limits). WMD: weighted mean difference.


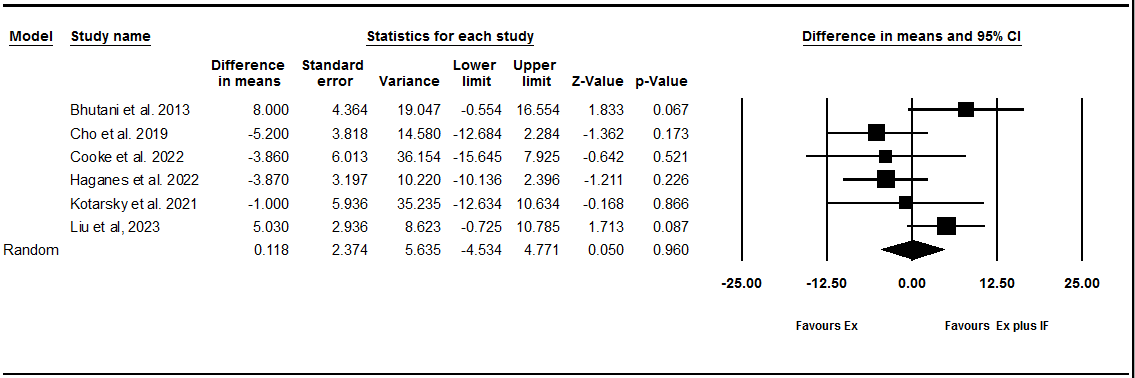
Supplementary Figure 16. Forest plot of the effects of Combined Ex and IF versus Ex alone on HDL. Data are reported as WMD (95% confidence limits). WMD: weighted mean difference.


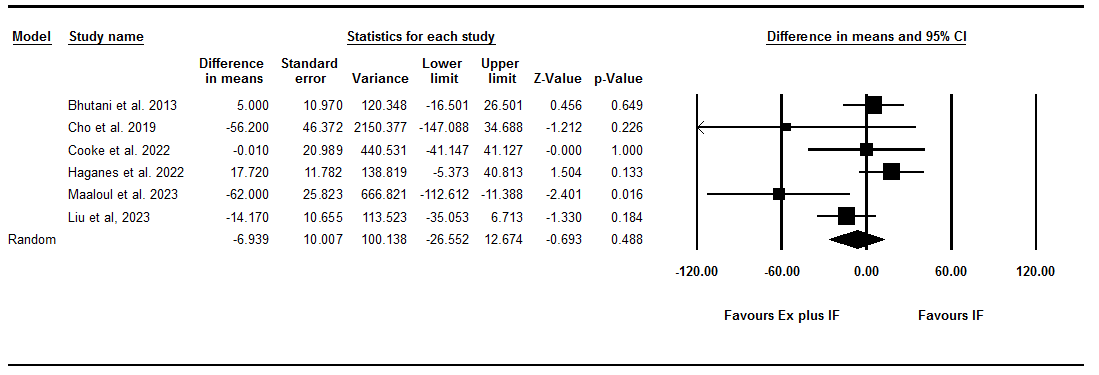
Supplementary Figure 17. Forest plot of the effects of Combined Ex and IF versus IF alone on TG. Data are reported as WMD (95% confidence limits). WMD: weighted mean difference.


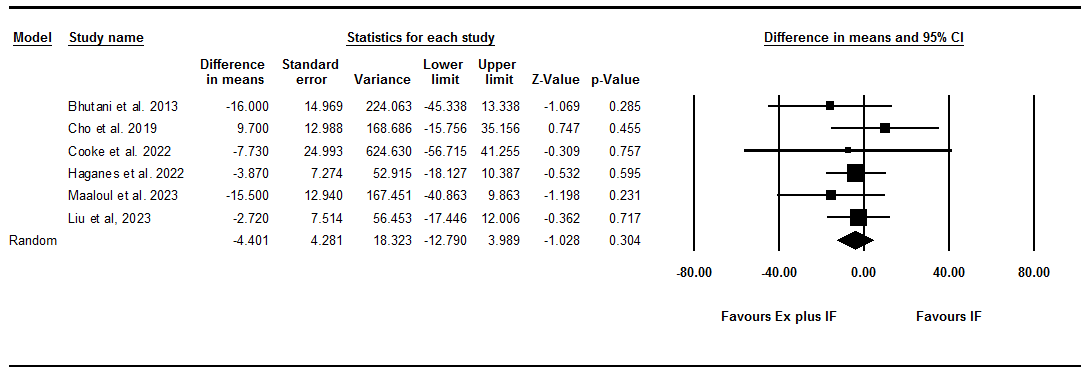
Supplementary Figure 18. Forest plot of the effects of Combined Ex and IF versus IF alone on TC. Data are reported as WMD (95% confidence limits). WMD: weighted mean difference.


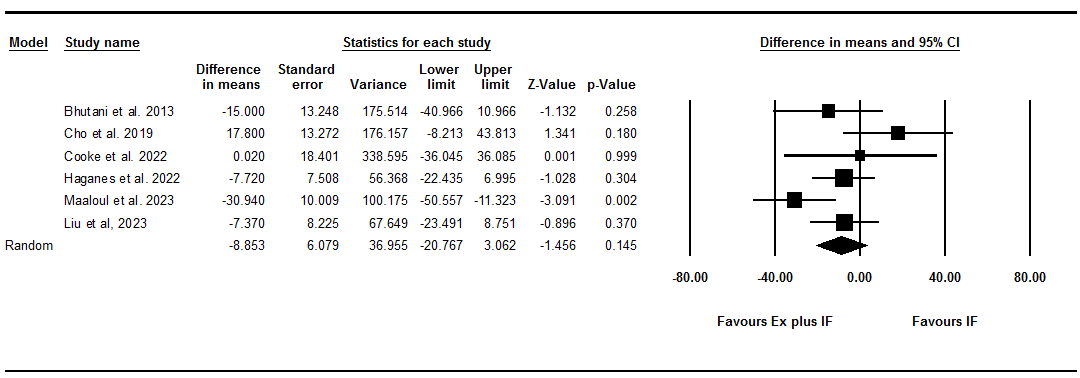
Supplementary Figure 19. Forest plot of the effects of Combined Ex and IF versus IF alone on LDL. Data are reported as WMD (95% confidence limits). WMD: weighted mean difference.


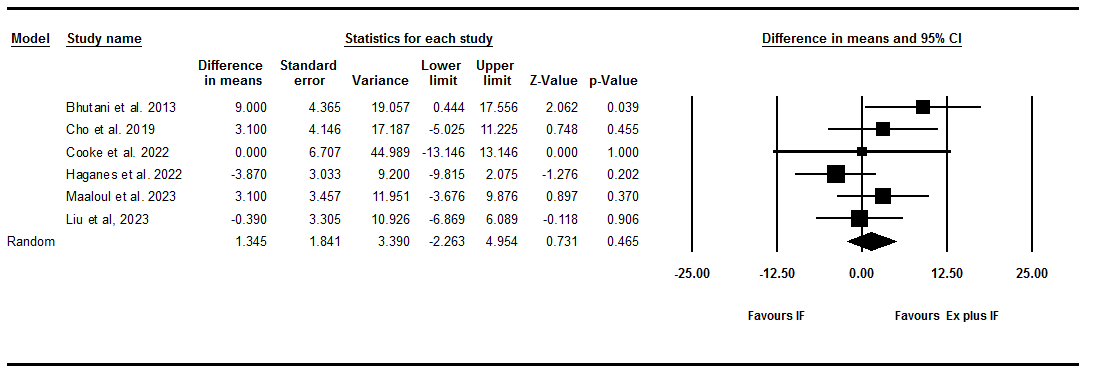
Supplementary Figure 20. Forest plot of the effects of Combined Ex and IF versus IF alone on HDL. Data are reported as WMD (95% confidence limits). WMD: weighted mean difference.


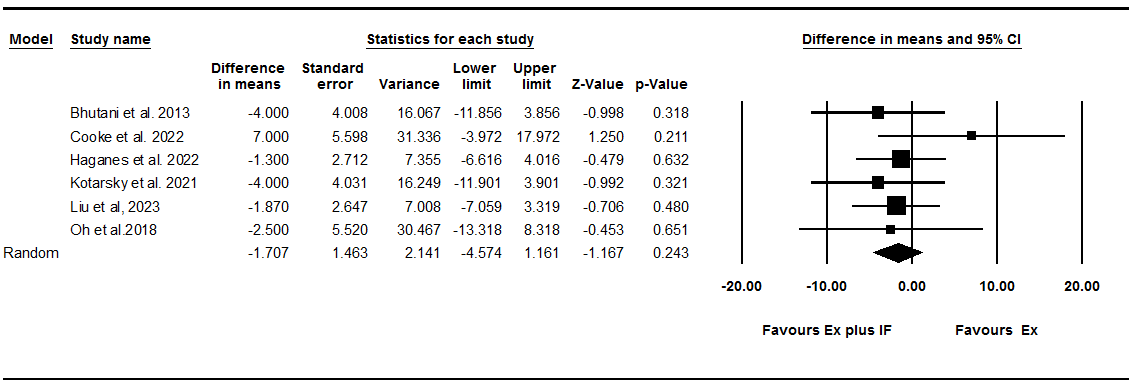
Supplementary Figure 21. Forest plot of the effects of Combined Ex and IF versus Ex alone on SBP. Data are reported as WMD (95% confidence limits). WMD: weighted mean difference.


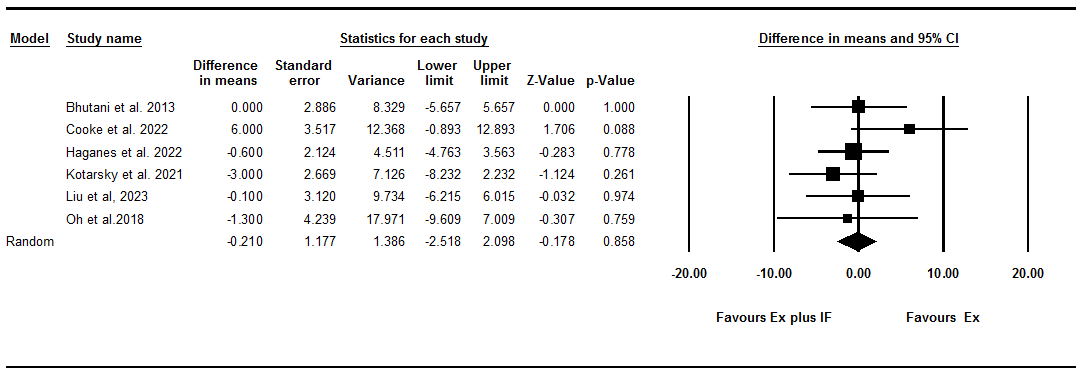
Supplementary Figure 22. Forest plot of the effects of Combined Ex and IF versus Ex alone on DBP. Data are reported as WMD (95% confidence limits). WMD: weighted mean difference.


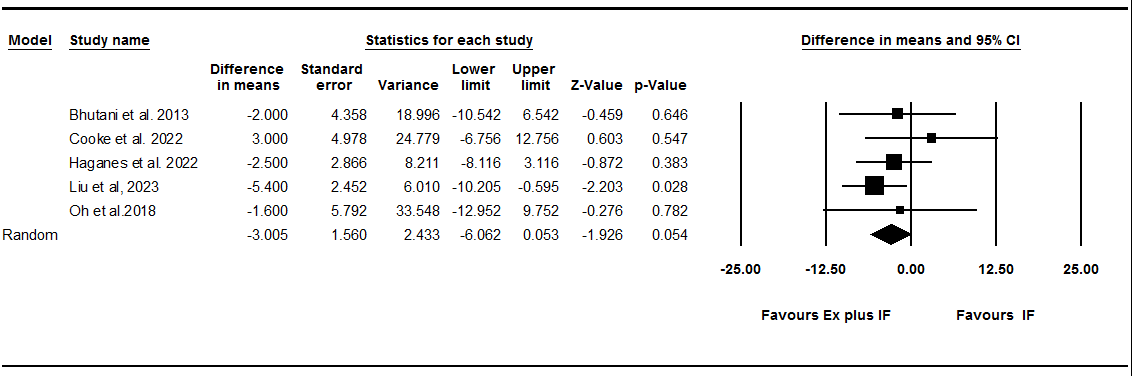
Supplementary Figure 23. Forest plot of the effects of Combined Ex and IF versus IF alone on SBP. Data are reported as WMD (95% confidence limits). WMD: weighted mean difference.


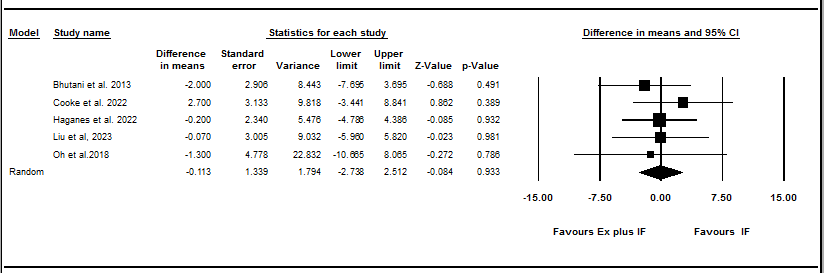
Supplementary Figure 24. Forest plot of the effects of Combined Ex and IF versus IF alone on DBP. Data are reported as WMD (95% confidence limits). WMD: weighted mean difference.


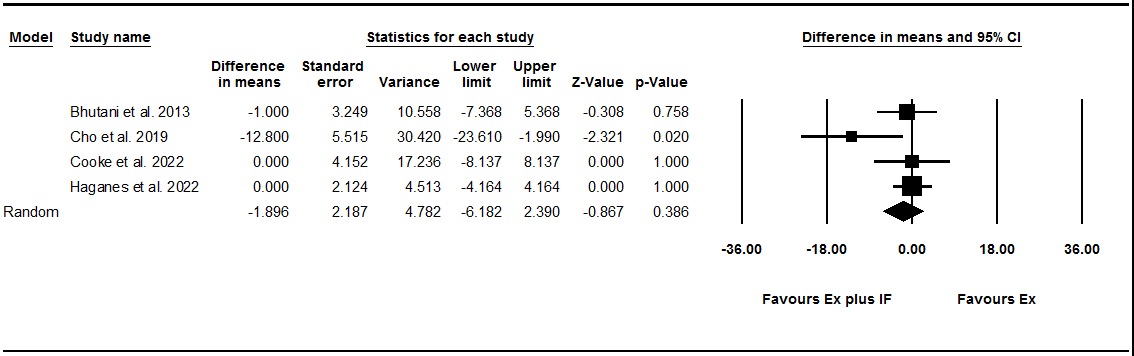
Supplementary Figure 25. Forest plot of the effects of Combined Ex and IF versus Ex alone on Glucose. Data are reported as WMD (95% confidence limits). WMD: weighted mean difference.


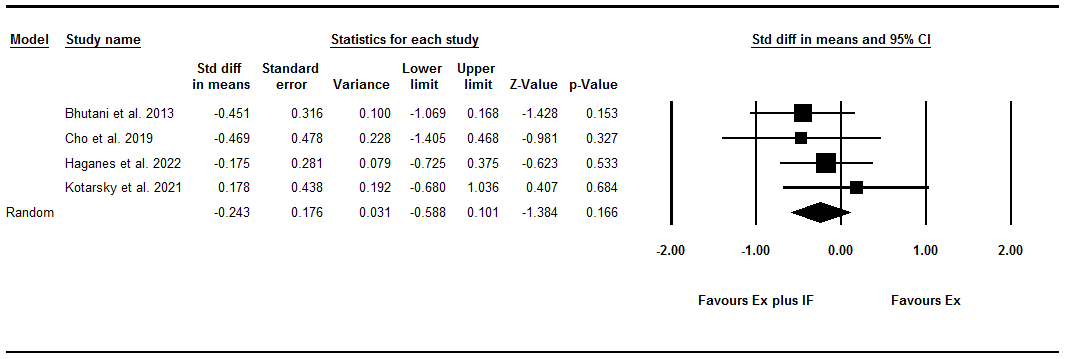
Supplementary Figure 26. Forest plot of the effects of Combined Ex and IF versus Ex alone on Insulin. Data are reported as SMD (95% confidence limits). SMD: Standardized mean difference.


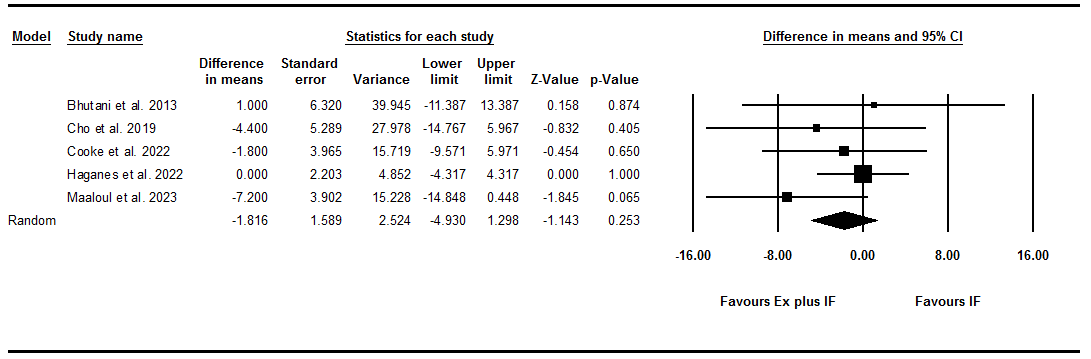
Supplementary Figure 27. Forest plot of the effects of Combined Ex and IF versus IF alone on Glucose. Data are reported as WMD (95% confidence limits). WMD: weighted mean difference.


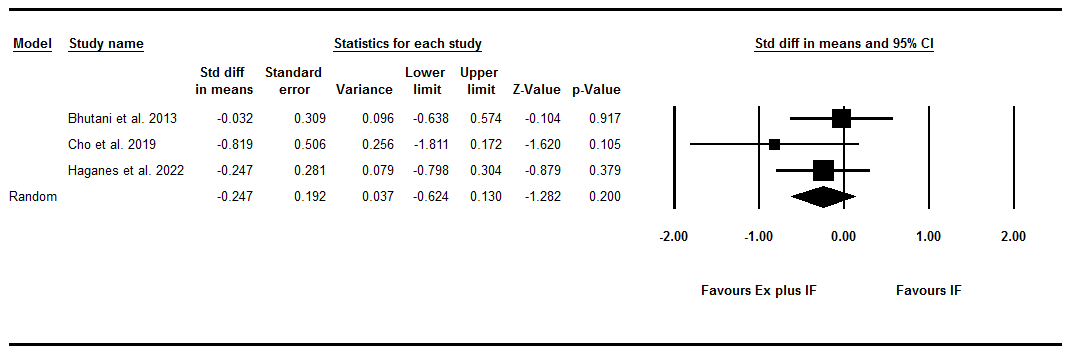
Supplementary Figure 28. Forest plot of the effects of Combined Ex and IF versus IF alone on Insulin. Data are reported as SMD (95% confidence limits). SMD: Standardized mean difference.


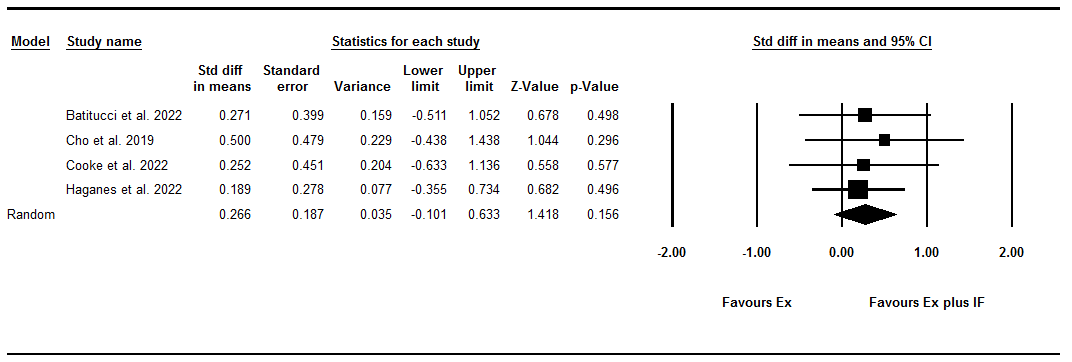
Supplementary Figure 29. Forest plot of the effects of Combined Ex and IF versus Ex alone on VO_2max/peck_. Data are reported as SMD (95% confidence limits). SMD: Standardized mean difference.


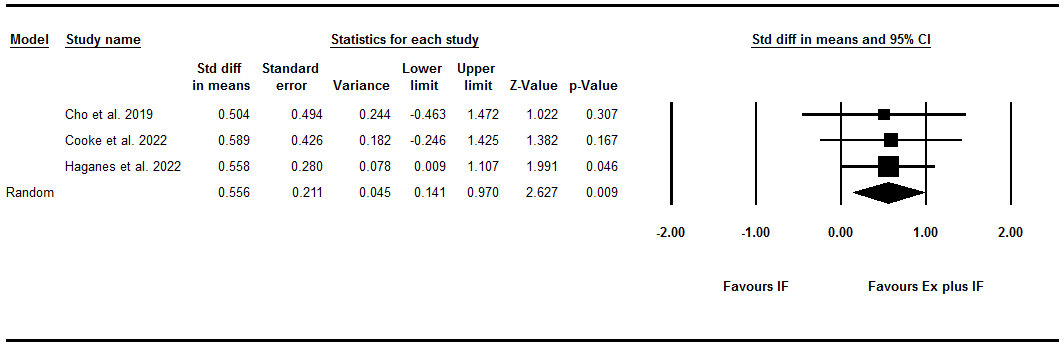
Supplementary Figure 30. Forest plot of the effects of Combined Ex and IF versus IF alone on VO_2max/peck_. Data are reported as SMD (95% confidence limits). SMD: Standardized mean difference.


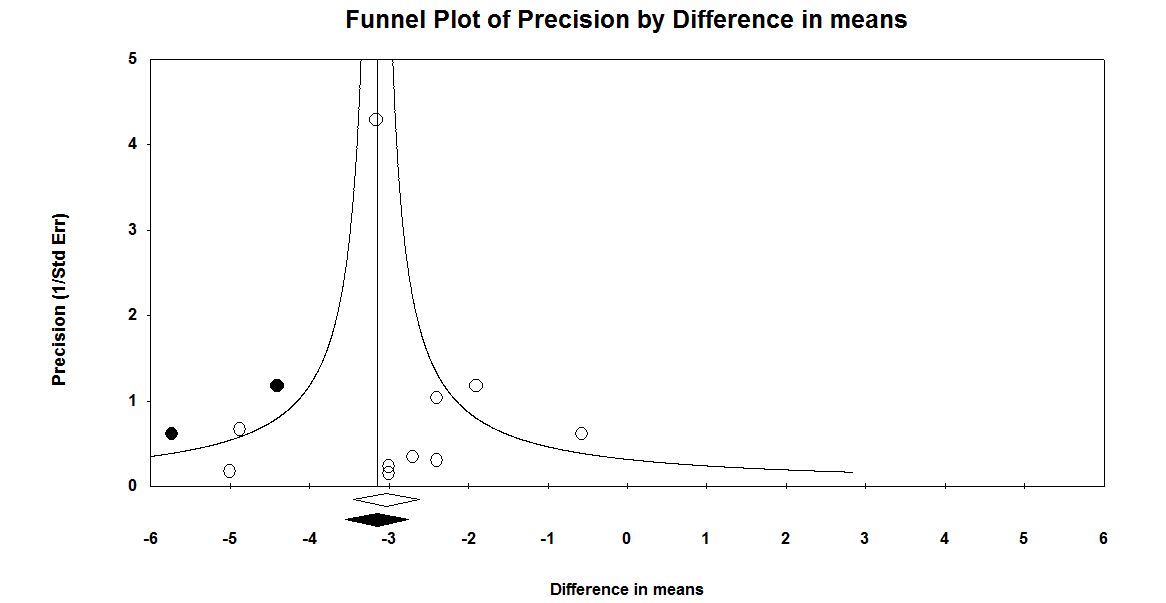
Supplementary Figure 31. Funnel plot of the effects of Combined Ex and IF versus Ex alone on Body weight.


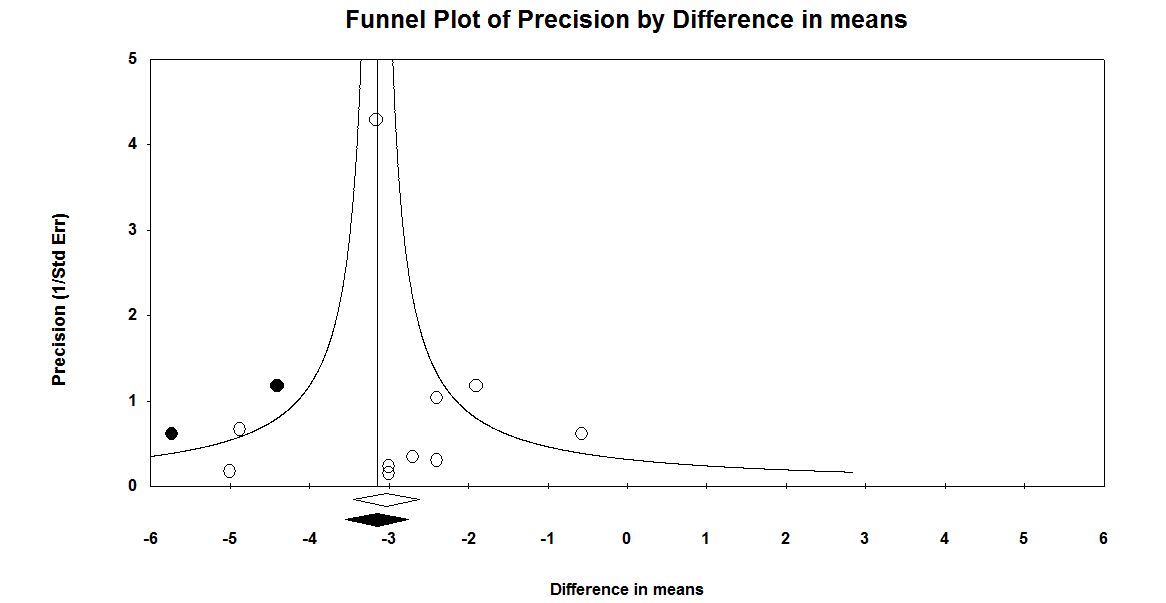
Supplementary Figure 32. Funnel plot of the effects of Combined Ex and IF versus Ex alone on BMI.


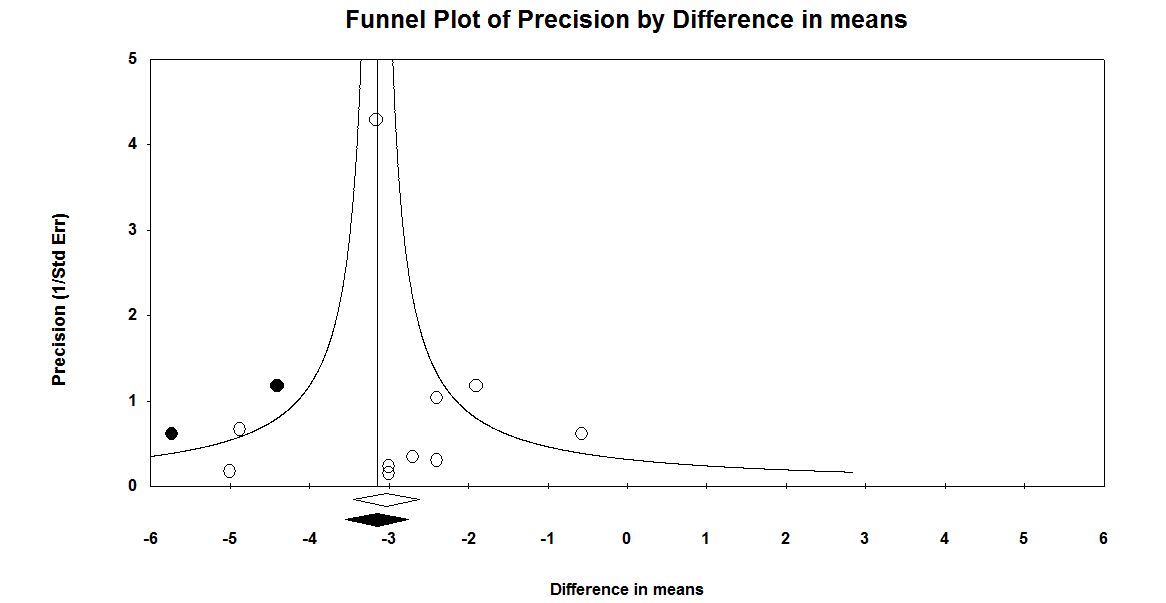
Supplementary Figure 33. Funnel plot of the effects of Combined Ex and IF versus Ex alone on Body fat.
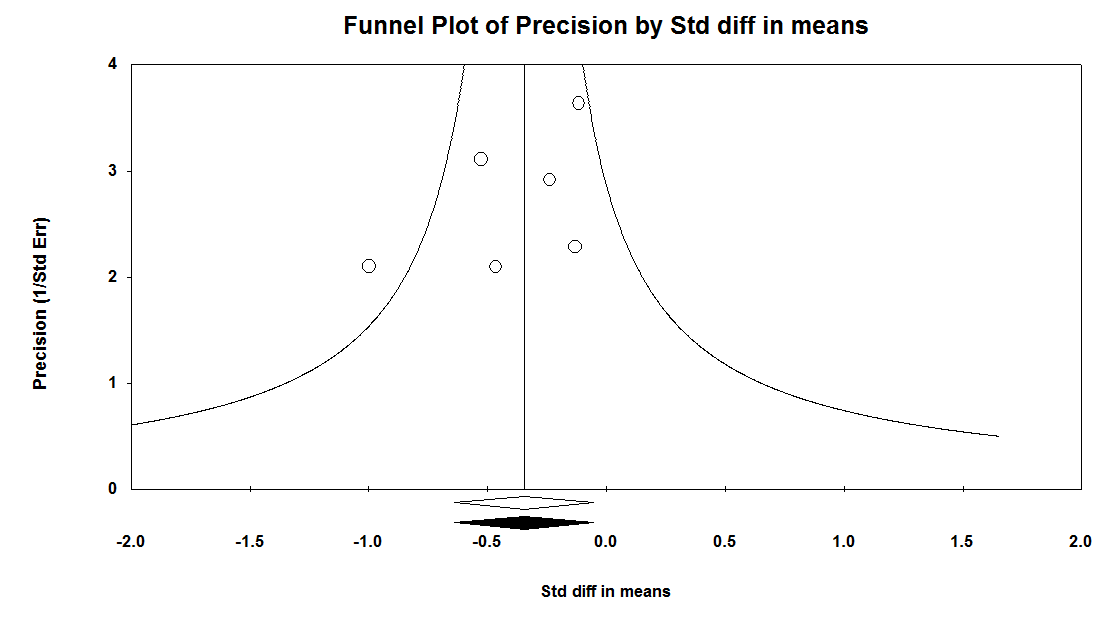
Supplementary Figure 34. Funnel plot of the effects of Combined Ex and IF versus Ex alone on Visceral fat.


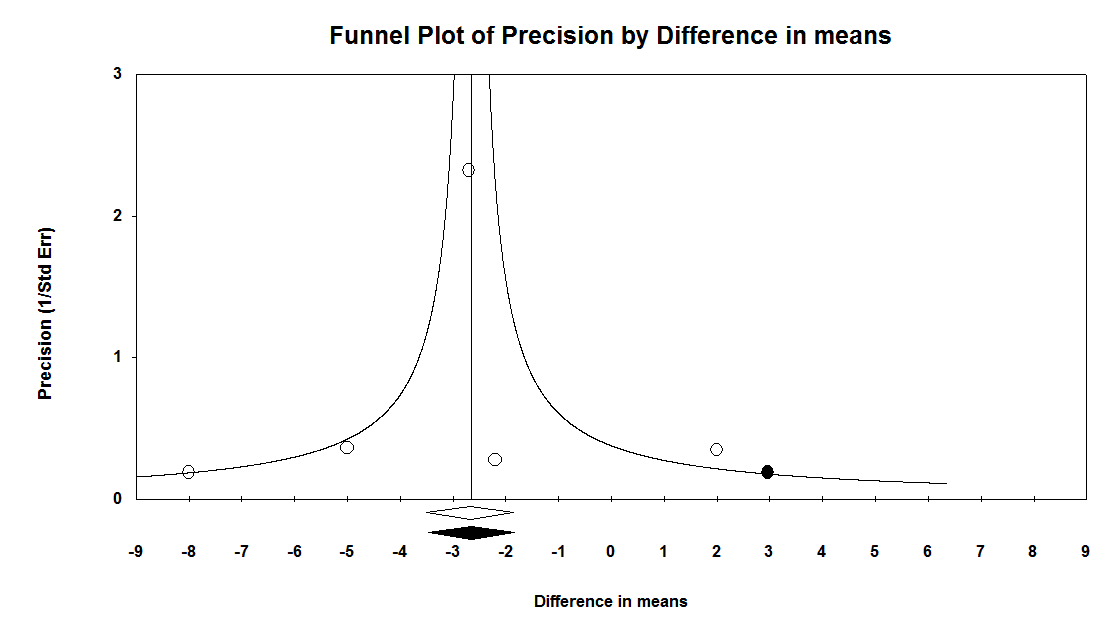
Supplementary Figure 35. Funnel plot of the effects of Combined Ex and IF versus Ex alone on waist circumference.


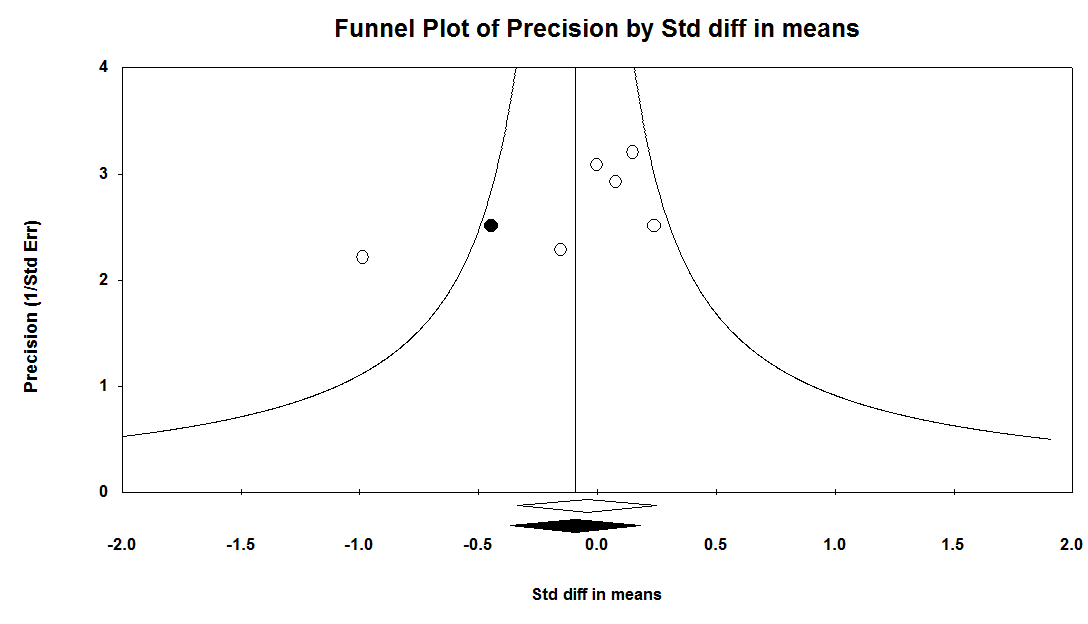
Supplementary Figure 36. Funnel plot of the effects of Combined Ex and IF versus Ex alone on LBM.


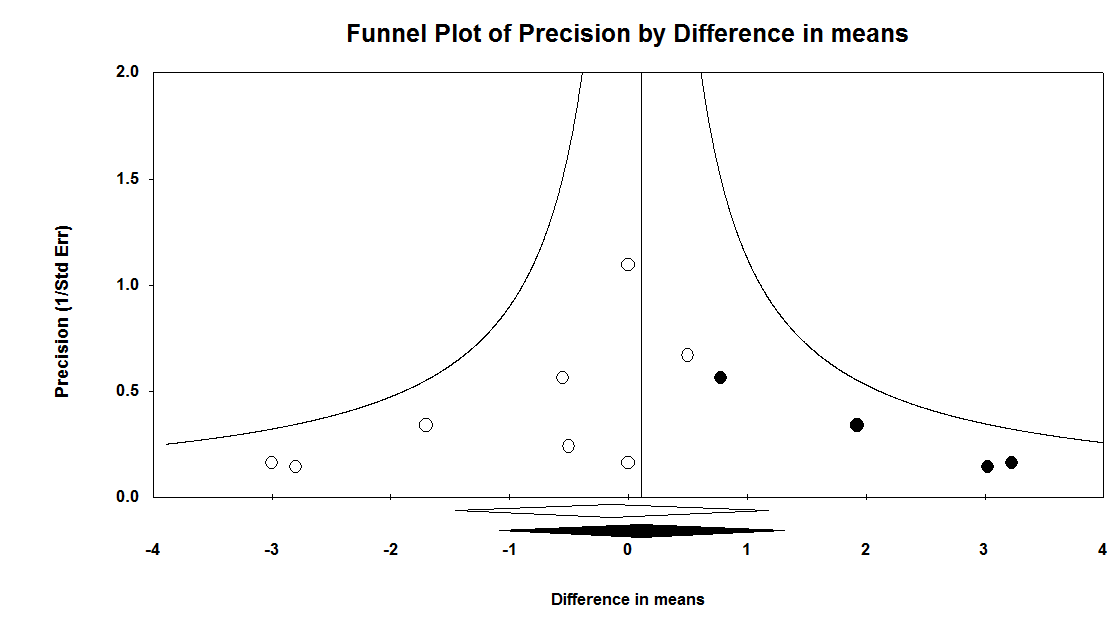
Supplementary Figure 37. Funnel plot of the effects of Combined Ex and IF versus IF alone on Body weight.


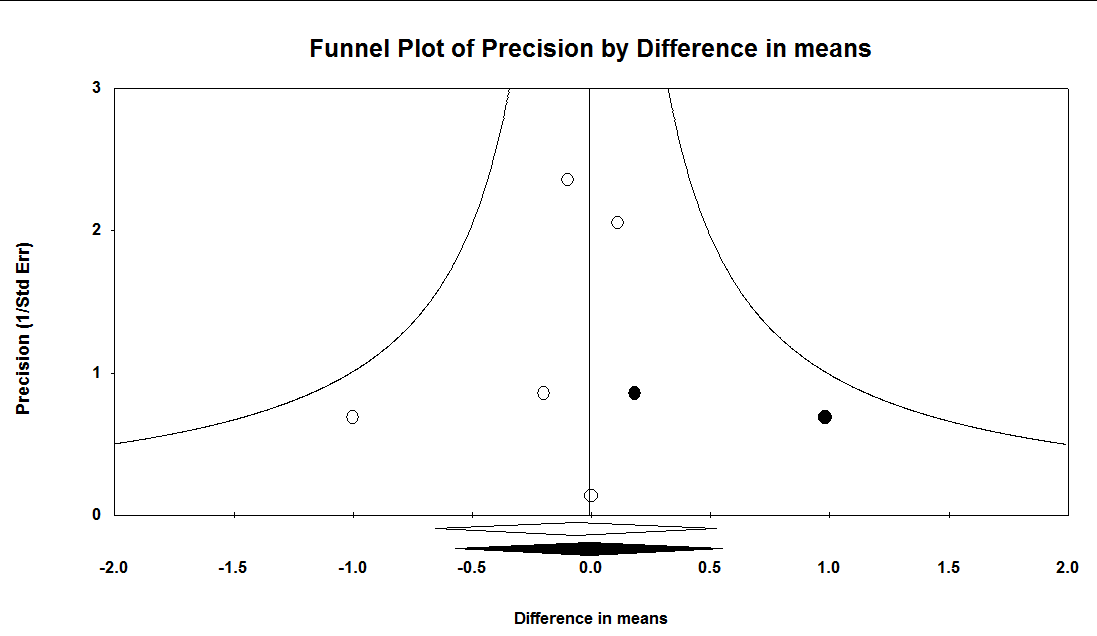
Supplementary Figure 38. Funnel plot of the effects of Combined Ex and IF versus IF alone on BMI.


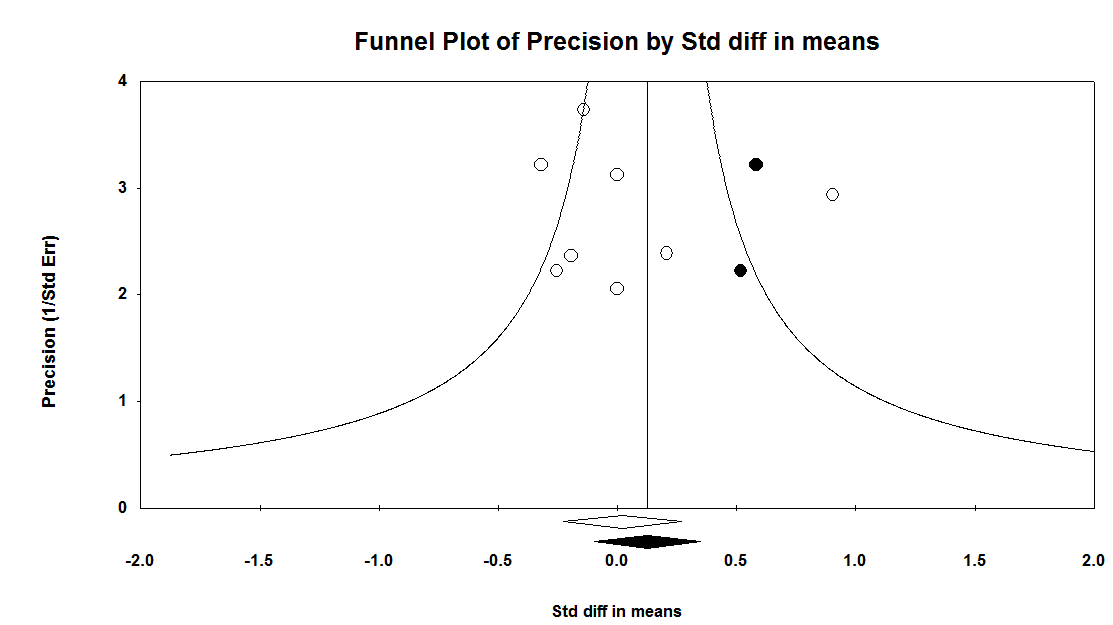
Supplementary Figure 39. Funnel plot of the effects of Combined Ex and IF versus IF alone on Body fat.


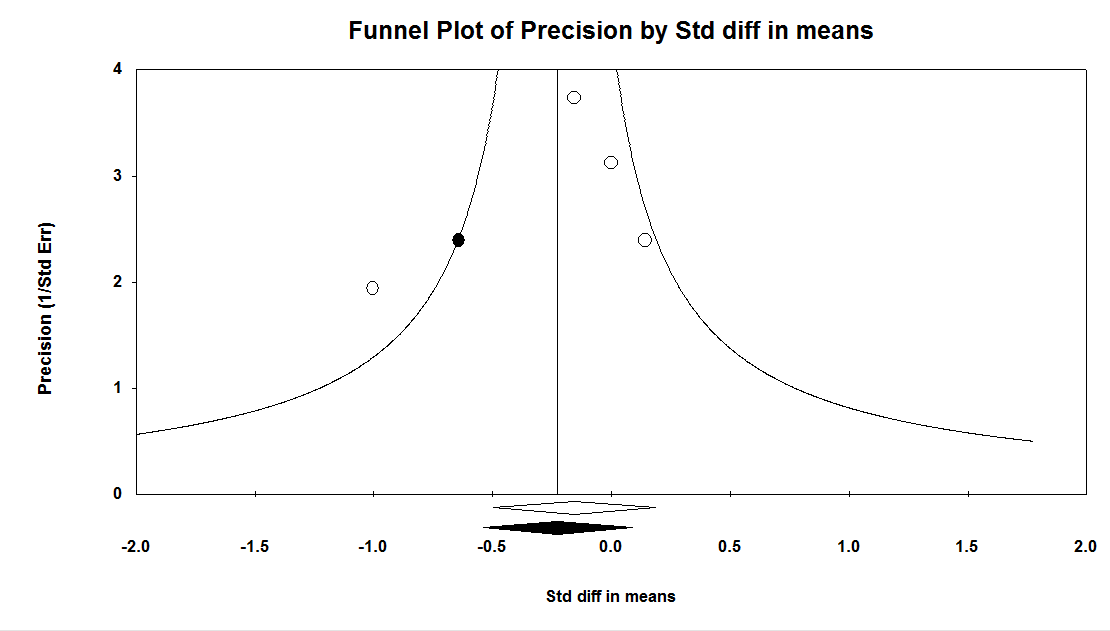
Supplementary Figure 40. Funnel plot of the effects of Combined Ex and IF versus IF alone on Visceral fat.


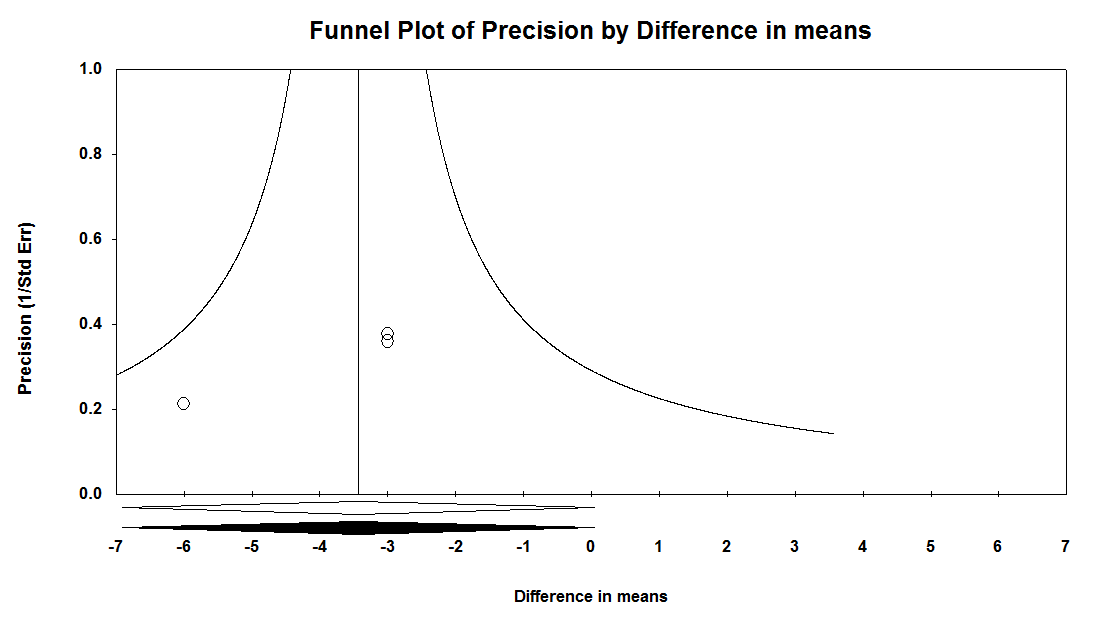
Supplementary Figure 41. Funnel plot of the effects of Combined Ex and IF versus IF alone on Waist circumference.


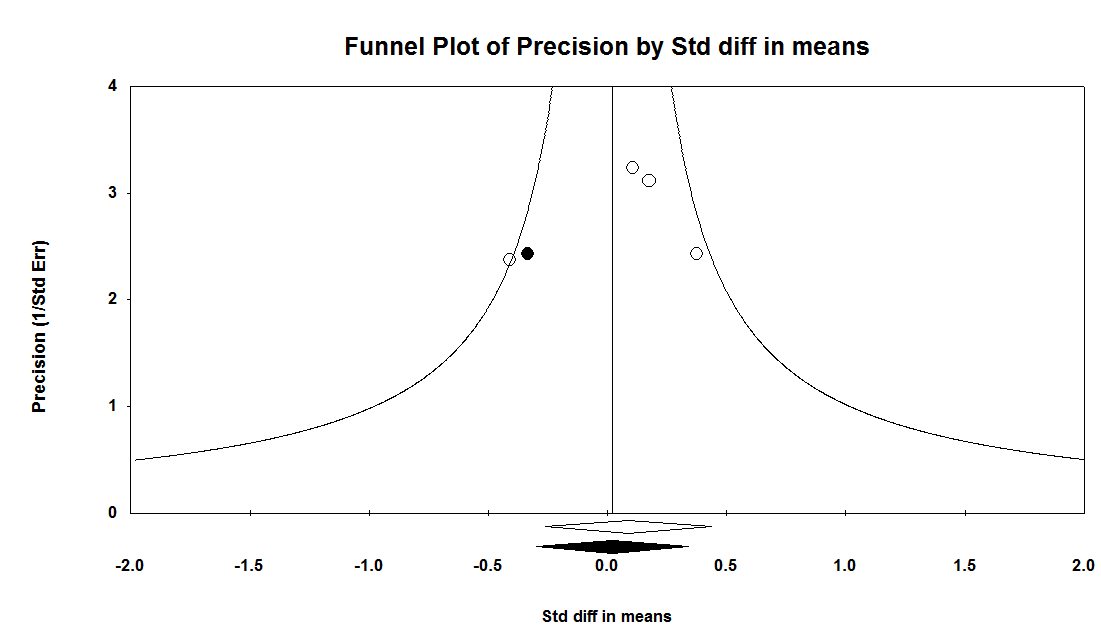
Supplementary Figure 42. Funnel plot of the effects of Combined Ex and IF versus IF alone on LBM.


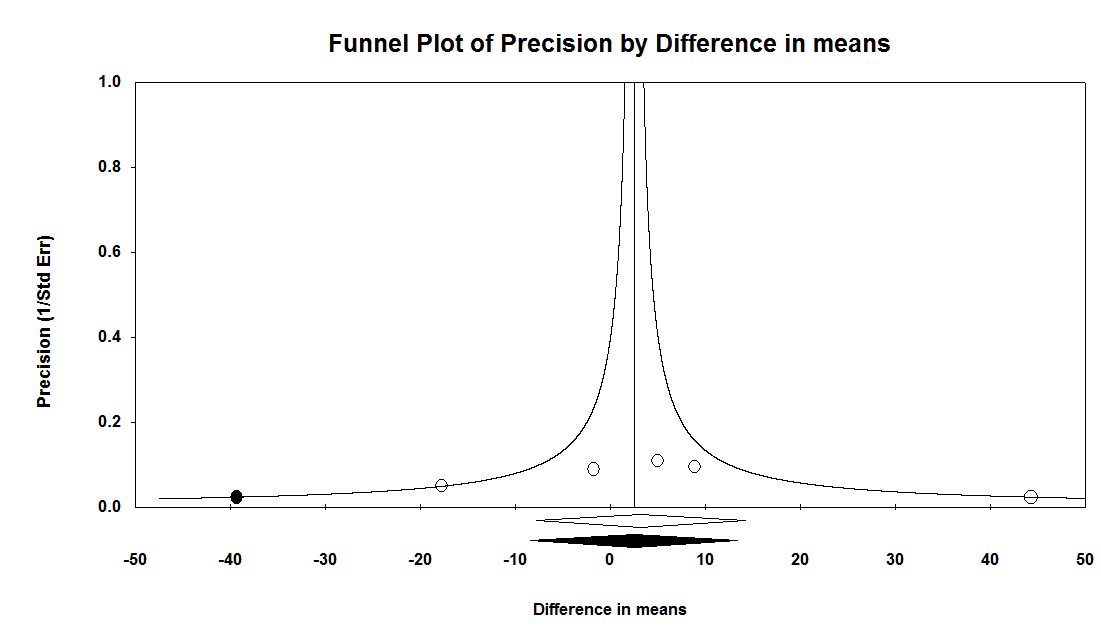
Supplementary Figure 43. Funnel plot of the effects of Combined Ex and IF versus Ex alone on TG.


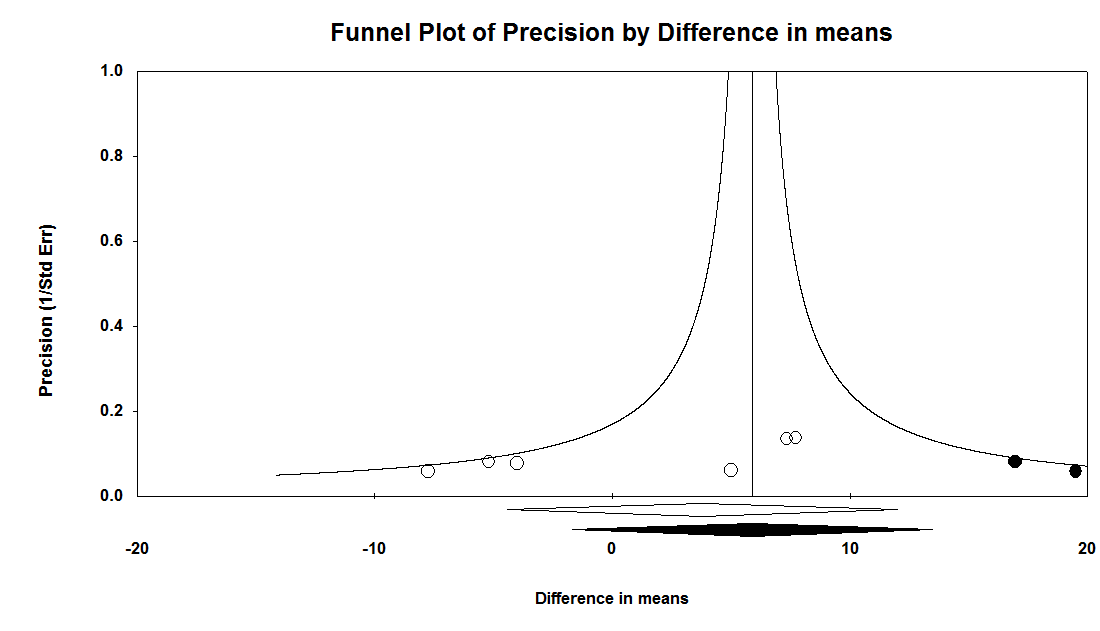
Supplementary Figure 44. Funnel plot of the effects of Combined Ex and IF versus Ex alone on TC.
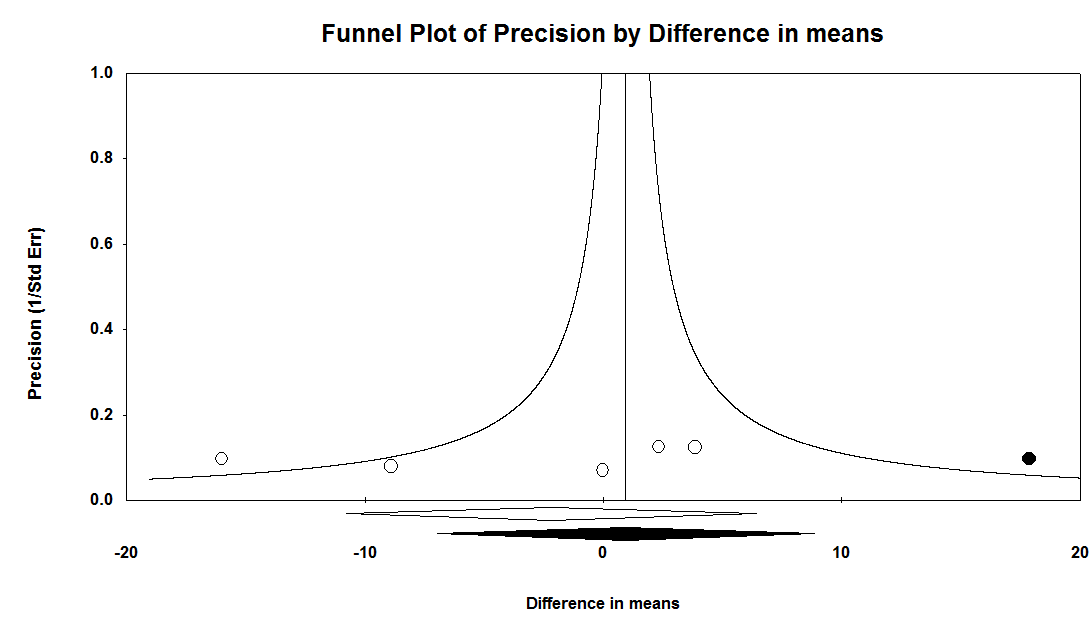
Supplementary Figure 45. Funnel plot of the effects of Combined Ex and IF versus Ex alone on LDL.


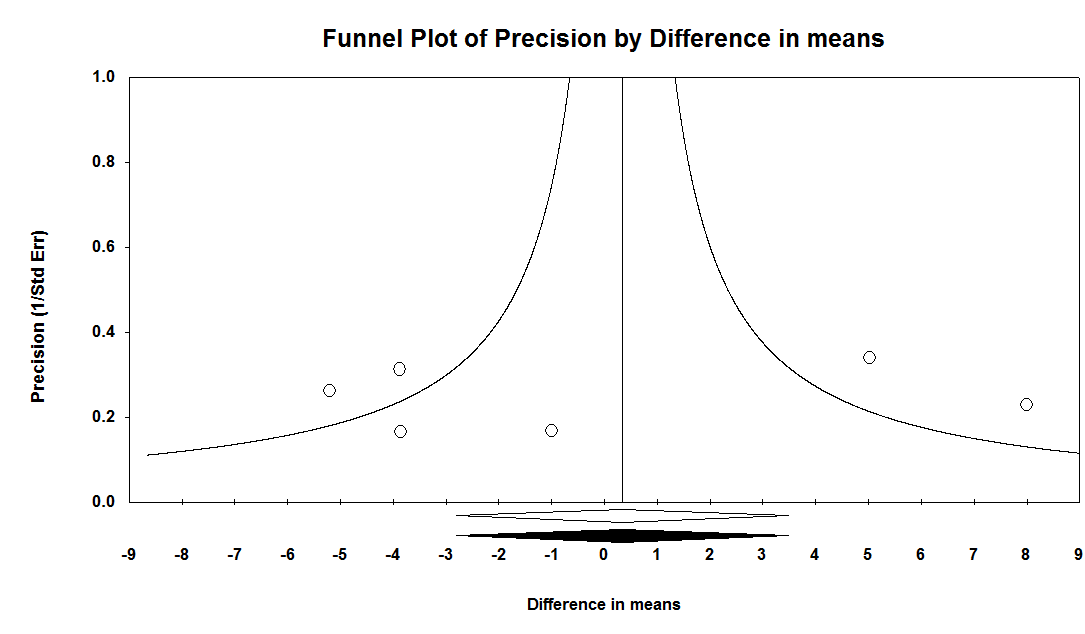
Supplementary Figure 46. Funnel plot of the effects of Combined Ex and IF versus Ex alone on HDL.


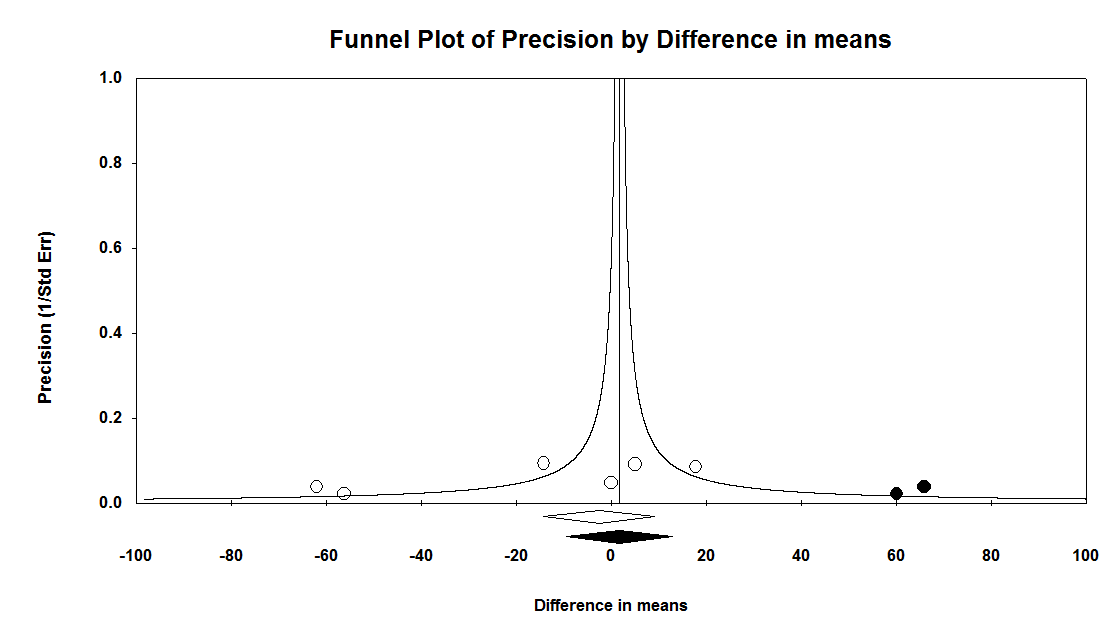
Supplementary Figure 47. Funnel plot of the effects of Combined Ex and IF versus IF alone on TG.


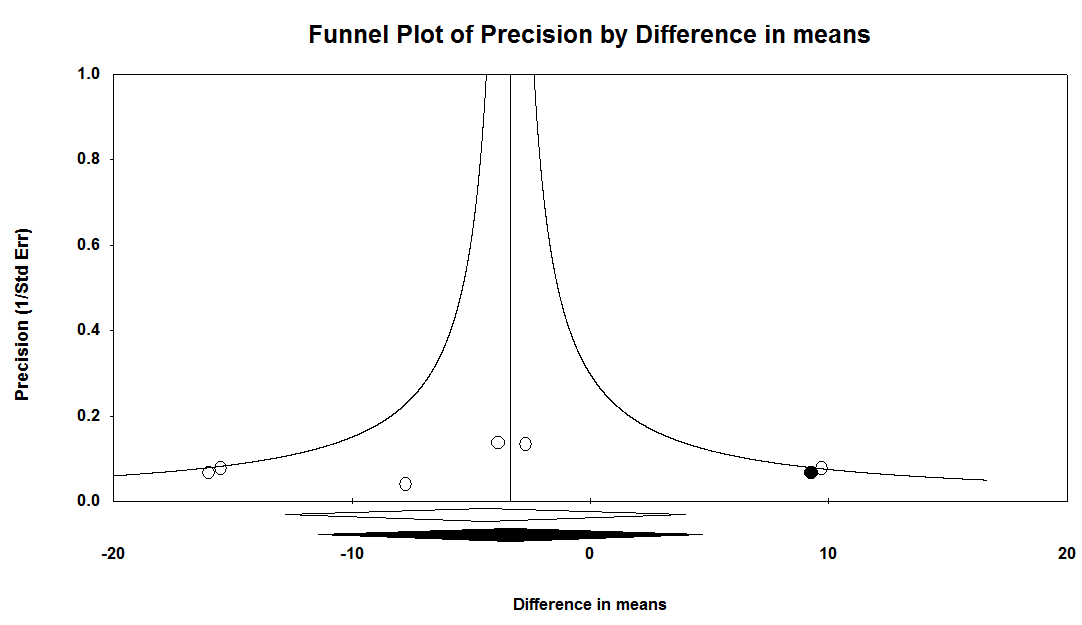
Supplementary Figure 48. Funnel plot of the effects of Combined Ex and IF versus IF alone on TC.


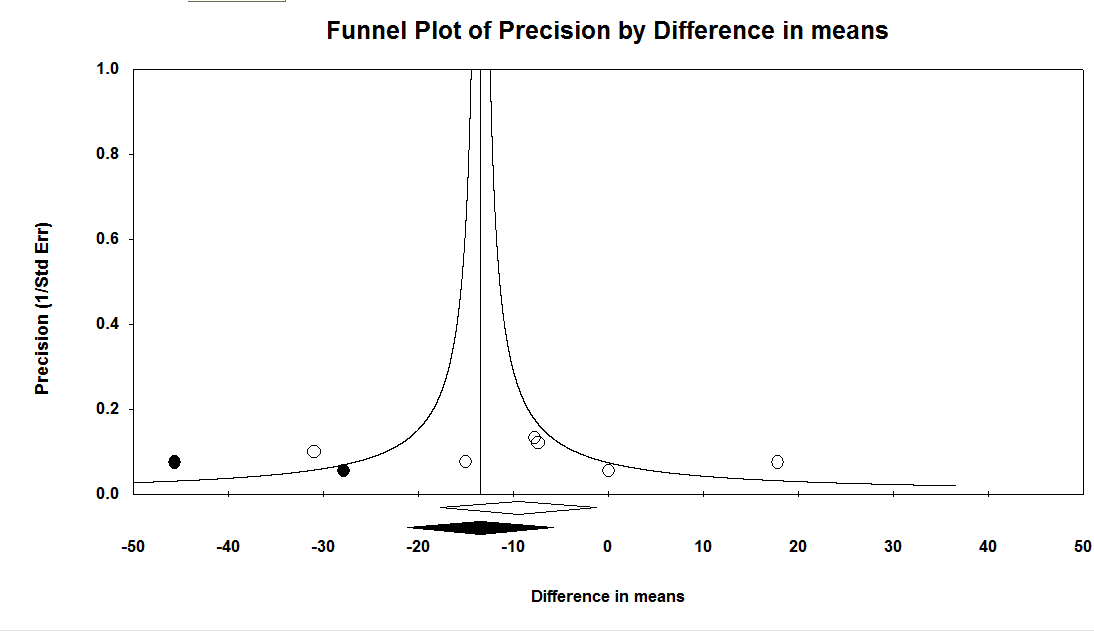
Supplementary Figure 49. Funnel plot of the effects of Combined Ex and IF versus IF alone on LDL.


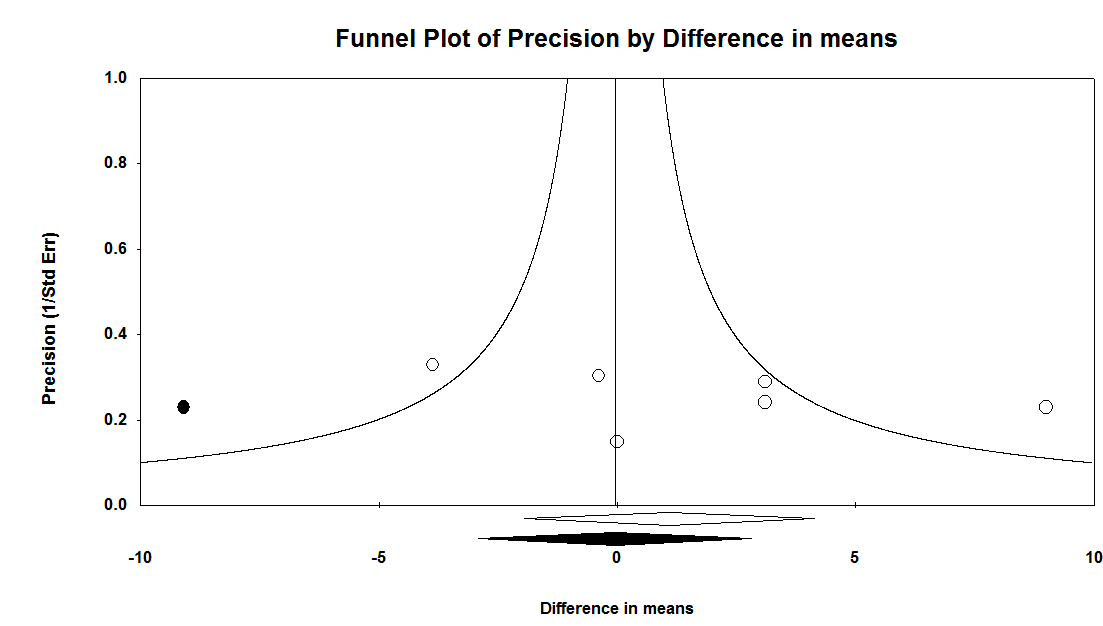
Supplementary Figure 50. Funnel plot of the effects of Combined Ex and IF versus IF alone on HDL.


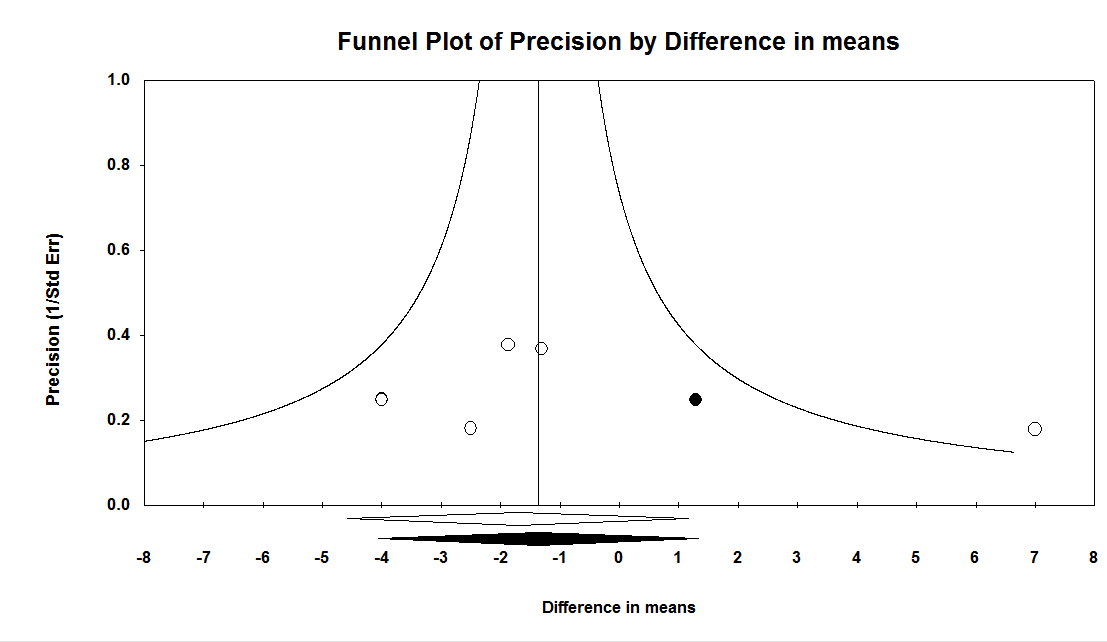
Supplementary Figure 51. Funnel plot of the effects of Combined Ex and IF versus Ex alone on SBP.


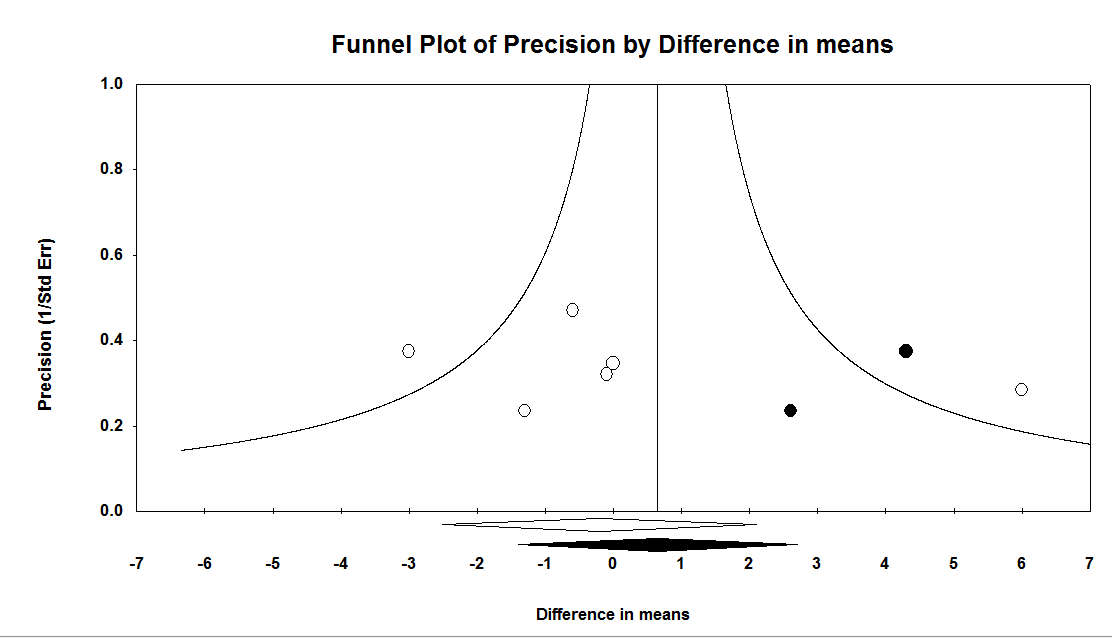
Supplementary Figure 52. Funnel plot of the effects of Combined Ex and IF versus Ex alone on DBP.


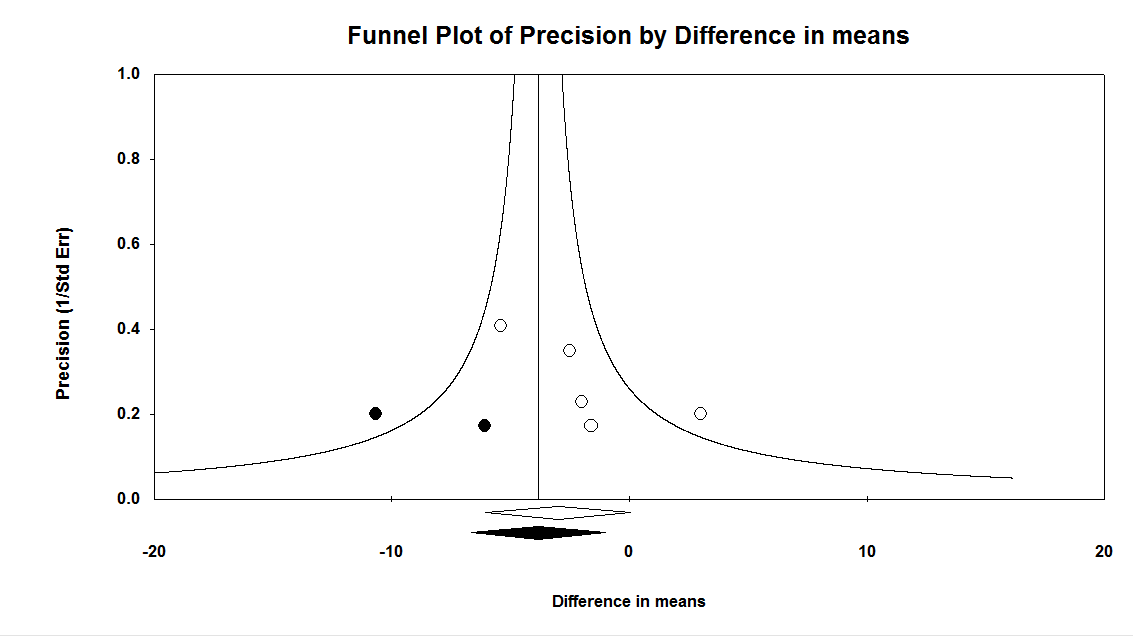
Supplementary Figure 53. Funnel plot of the effects of Combined Ex and IF versus IF alone on SBP.


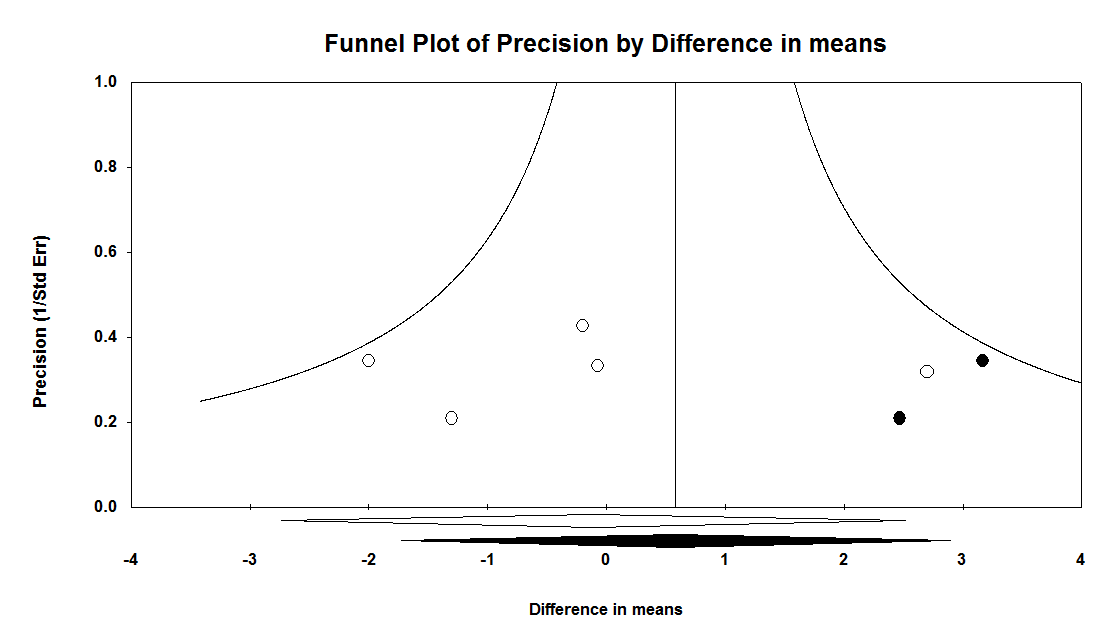
Supplementary Figure 54. Funnel plot of the effects of Combined Ex and IF versus IF alone on DBP.


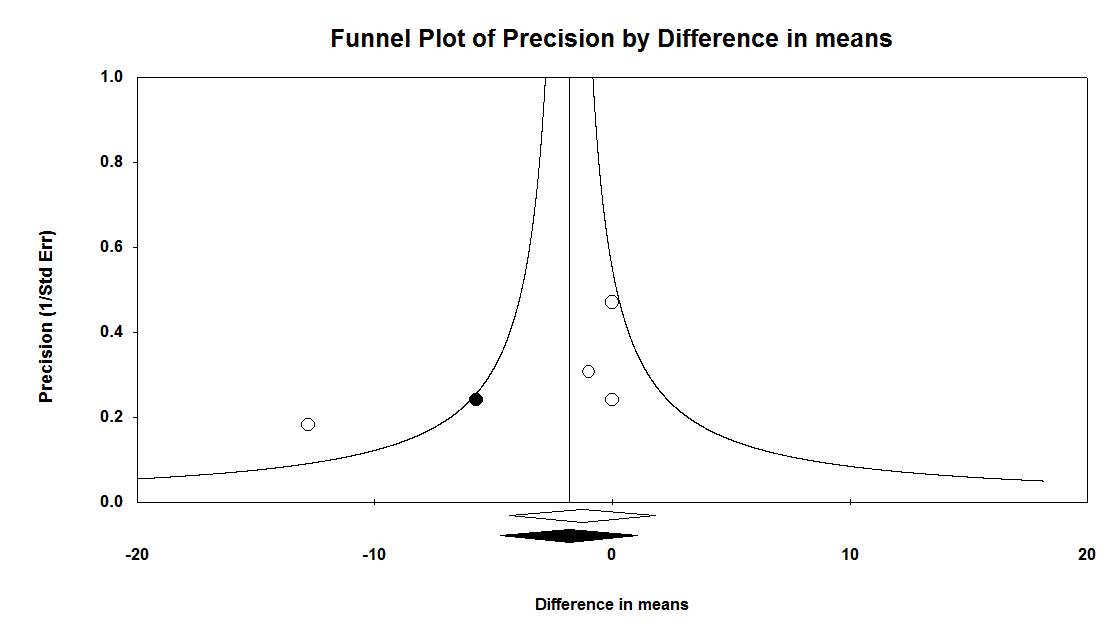
Supplementary Figure 55. Funnel plot of the effects of Combined Ex and IF versus Ex alone on Glucose.
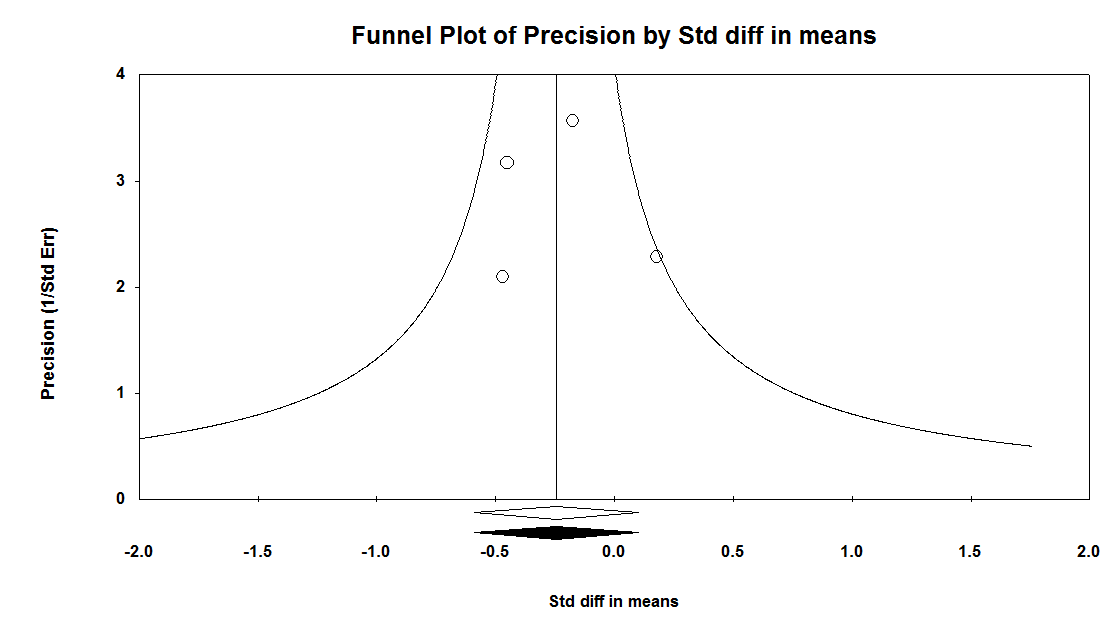
Supplementary Figure 56. Funnel plot of the effects of Combined Ex and IF versus Ex alone on Insulin.


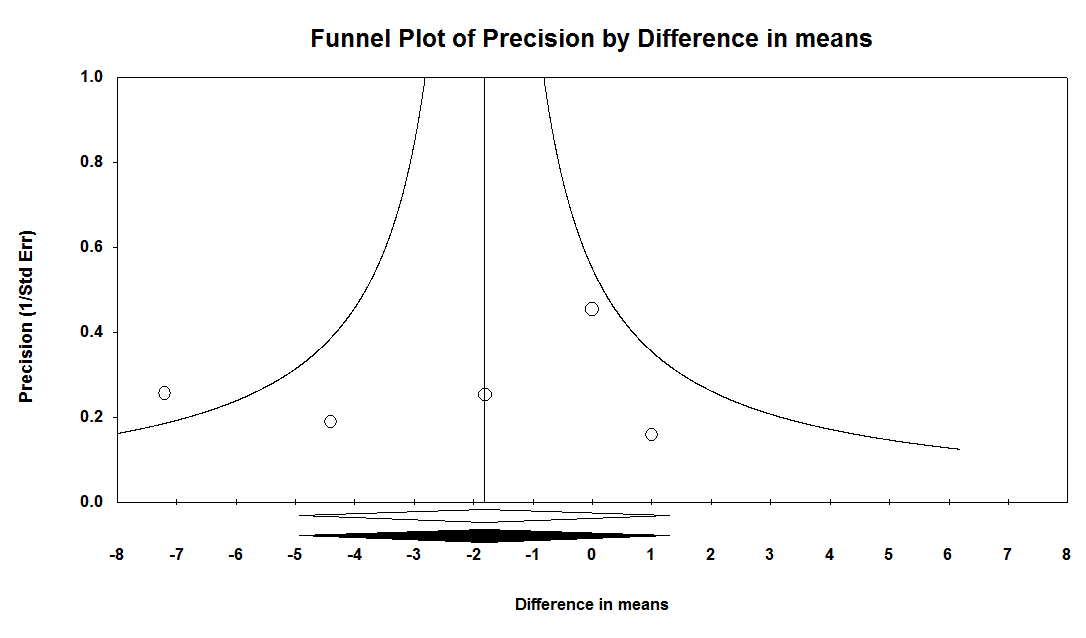
Supplementary Figure 57. Funnel plot of the effects of Combined Ex and IF versus IF alone on Glucose.


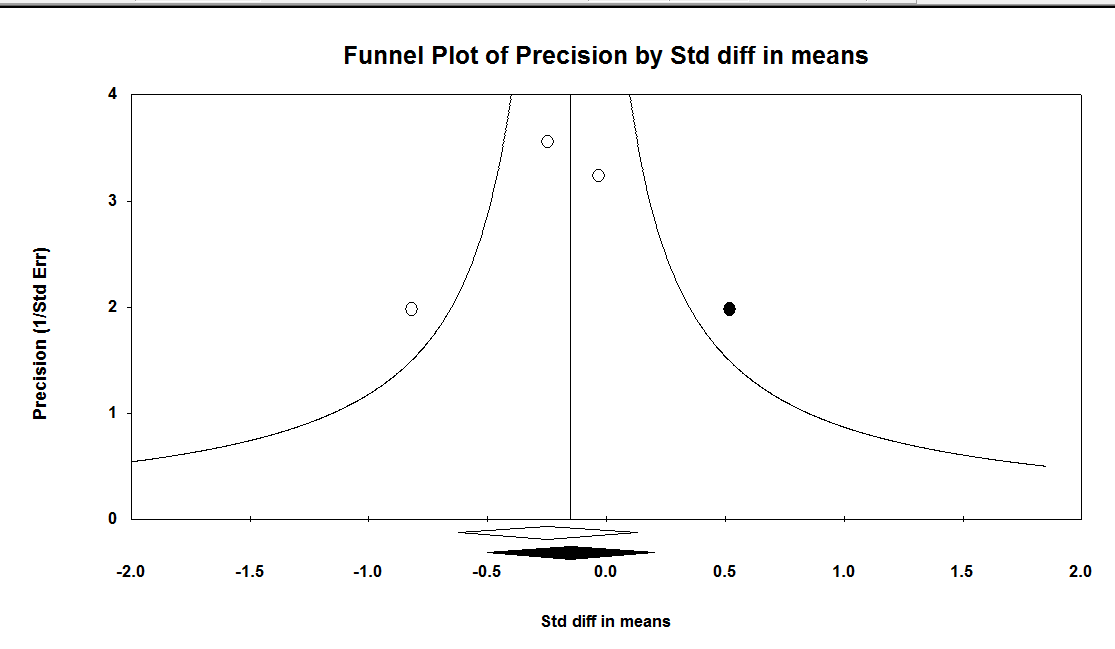
Supplementary Figure 58. Funnel plot of the effects of Combined Ex and IF versus IF alone on Insulin.


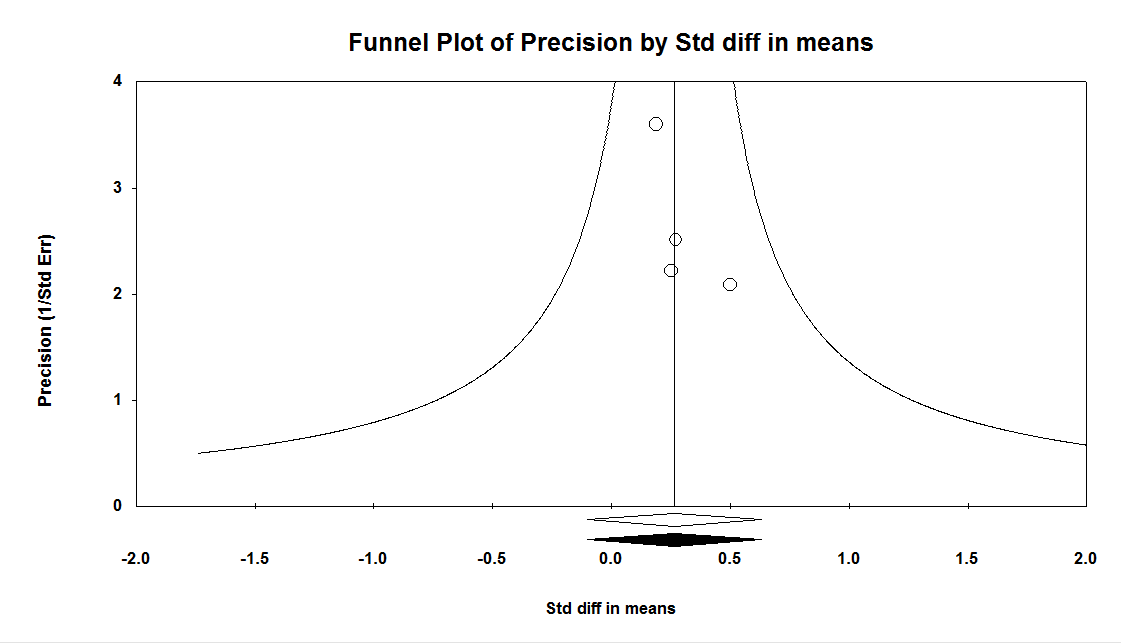
Supplementary Figure 59. Funnel plot of the effects of Combined Ex and IF versus Ex alone on VO_2max/peck_.


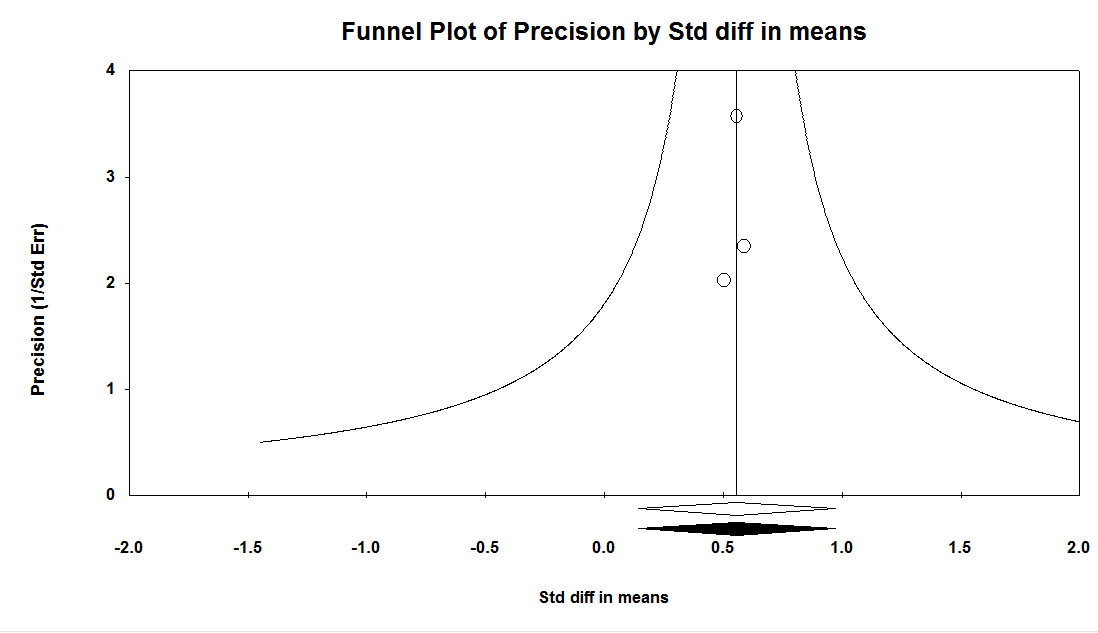
 Supplementary Figure 60. Funnel plot of the effects of Combined Ex and IF versus IF alone on VO_2max/peck_.
